# Structural Distortions and Uniaxial Negative Thermal Expansion in the Polar Dion–Jacobson Oxide RbNdTa_2_O_7_

**DOI:** 10.1021/acs.chemmater.5c00137

**Published:** 2025-05-15

**Authors:** P. Neenu Lekshmi, E. Lora da Silva, P. Rocha-Rodrigues, John S. O. Evans, João Horta Belo, Pedro Silva de Sousa, Alicia María Manjón-Sanz, António M. dos Santos, Armandina M. L. Lopes, João Pedro Araújo

**Affiliations:** † IFIMUP, Institute of Physics for Advanced Materials, Nanotechnology and Photonics, Department of Physics and Astronomy, Faculty of Sciences, 615871 University of Porto, Rua do Campo Alegre, 687, 4169-007 Porto, Portugal; ‡ High Performance Computing Chair, 16774 University of Évora, Rua Romão Ramalho 59, 7000-671 Évora, Portugal; § Department of Chemistry, University Science Site, 3057 Durham University, South Road, Durham, DH1 3LE, U.K.; ∥ Neutron Scattering Division, 6146 Oak Ridge National Laboratory, Oak Ridge, Tennessee 37831, United States

## Abstract

We provide deeper insight into the crystal structures, sequential structural phase transitions (*I2cm* → *Cmce* → *I*4/*mcm* → *P*4/*mmm*), thermal expansion, and electronic properties of the *n* = 2 Dion–Jacobson polar oxide RbNdTa_2_O_7_, through X-ray powder diffraction, neutron powder diffraction, Raman studies, and density functional theory calculations. We observed a uniaxial negative thermal expansion (NTE) across the first-order transition, *I2cm* → *Cmce*, where the unit cell contracts along the *c-*axis, which is driven by a contraction of the NdTa_2_O_6_ layer. This NTE occurs within the temperature range of the first-order phase transition and contrasts with the corkscrew mechanism typically observed in Ruddlesden–Popper phases. In RbNdTa_2_O_7_, the *I2cm* (hybrid improper ferroelectric) → *Cmce* (antipolar) transition involves crucial changes in the bond lengths of Nd and Ta polyhedra, coupled with polar to antipolar displacement of the Nd ions, leading to a net contraction in the NdTa_2_O_6_ layer along the *c-*axis, while preserving the overall octahedral tilting magnitude. This transition highlights the intricate interplay between the Nd and Ta coordination and the associated TaO_6_ distortions. Temperature-dependent Raman spectra analysis further confirms the first-order structural transition and associated NTE, providing evidence for increased bond stiffness across this transition. Additionally, using neutron powder diffraction, we have determined that the transition *I*4/*mcm* → *P*4/*mmm* occurs at approximately 1150 K. Finally, we have calculated from DFT + *U*, the partial density of states, the energy bandgaps, and effective masses of the charge carriers of the polar ground structure.

## Graphical abstract

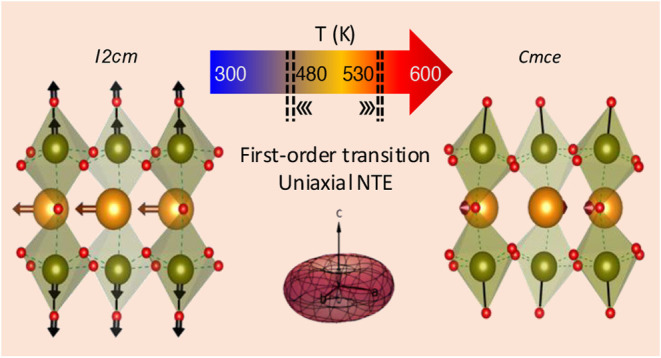

## Introduction

Understanding and manipulating the lattice distortions in complex perovskite oxides captivate chemists, physicists, and materials scientists, since it is crucial for designing and uncovering technologically important functional materials. Structural distortions where inversion symmetry breaking occurs are particularly interesting because spontaneous and switchable electrical polarization can be induced. Such effects have important applications for several devices, spanning from capacitors and nonvolatile memories, to sensors and transducers, as well as energy harvesting materials and beyond.^1^ In perovskites, with structural formula ABO_3_, ferroelectricity can be either induced by second-order Jahn–Teller (SOJT) distortion (due to charge transfer or hybridization between different electronic states, as observed in BaTiO_3_ or BiFeO_3_), or through geometrically induced distortions (due to electrostatic and size effects of cations, as found for YMnO_3_).^2–5^

A novel mechanism of ferroelectricity, known as hybrid improper ferroelectricity (HIF), has been elucidated in perovskite-related layered oxides, such as Ruddlesden–Popper (RP) and Dion–Jacobson (DJ) structures.^6–9^ The RP, A_*n*+1_B_*n*_O_3*n*+1_, and DJ, A′A_*n*–1_B_*n*_O_3*n*+1_, families of oxides consist of perovskite slabs composed of *n* layers of cornershared BO_6_ octahedra sandwiched between other metal oxide layers to form a natural superlattice. The displacement of the alternating perovskite slabs is usually 1/2 [110] for RP. For DJ, the displacement can be either 1/2 [100/010] or absent, depending on the size of A′cations.^7^ Compared to the ABO_3_ perovskites, the layered derivatives possesses additional symmetry breaking due to the combined effect of layering and cooperative octahedral tilts.^9,10^ The HIF mechanism involves trilinear coupling of two nonpolar tilting distortions together with a polar distortion mode. Beyond the HIF behavior, layered perovskites showcase distinct photoelectric properties and tunable electronic band structures, offering significant potential for photocatalysis, photoelectrochemical water splitting, and photoluminescence applications.^11–15^ The combination of ferroelectricity and superior optical-electronic properties in layered oxides also suggests the feasibility of fabricating materials with the possibility for photoferroic effect.^16^

The polar HIF mechanism among the DJ oxides was first proposed by Benedek^7^ through a computational study on *n* = 2 DJ oxides such as A′ANb_2_O_7_ (A′ = Rb, Cs; A = Y, La, Nd, Bi), by using first-principles density functional theory together with symmetry group-subgroup analysis and crystal-chemical models. Among those materials, A′BiNb_2_O_7_ (A′ = Rb, Cs) and CsNdNb_2_O_7_ were previously observed, via experimental techniques, to evidence a ground-state polar phase.^17,18^ However, for A′LaNb_2_O_7_ (A′ = Rb, Cs), RbNdNb_2_O_7_, as well as for similar structured tantalates, such as A′ATa_2_O_7_ (A′ = Cs, Rb; A = La, Pr, Nd, and Sm), nonpolar structural phases had instead been experimentally observed,^11,19,20^ prompting a reevaluation of these materials. Subsequently, Zhu et al.,^21,22^ by employing an integrated theoretical-experimental approach, confirmed the presence of polar ground states for *n* = 2 DJ oxides, specifically for A′NdM_2_O_7_ (A′ = Rb, Cs; M = Nb, Ta).

Combined experimental techniques and theoretical simulations are used to probe the structural, electronic and vibrational features of layered oxides with HIF.^9,10,23,24^ According to Zhu et al.,^21,22^ RbNdM_2_O_7_ and CsNdM_2_O_7_ adopt distinct polar ground state structures with *I2cm* and *P2*_1_ *am* symmetries, respectively. In both compounds, the lowest-energy structures exhibit a trilinear coupling. However, the Rb phases display more complex tilting modes, with the tilting distortion alternating between neighboring layers, *a* ^–^ *a* ^–^ *c* ^+^ */*–*(a* ^–^ *a* ^–^ *c* ^+^ *)*. Furthermore, both compounds undergo sequential structural transitions, culminating in a centrosymmetric *P*4/*mmm* phase at high temperatures. The phase transition temperature to *P*4/*mmm* is 800 K for CsNdNb_2_O_7_, 820 K for CsNdTa_2_O_7_, and yet to be identified for RbNdM_2_O_7_, however, the *P*4/*mmm* phase has been experimentally observed at 1273 K.^21^ Recent studies on similar DJ oxides, including the experimental confirmation of polar structures in RbPrNb_2_O_7_ ^25^ and A′SmNb_2_O_7_ (A′ = Rb, Cs);^26^ structural frustration effects in (Cs,Rb)­NdNb_2_O_7_;^27^ and the suppression of ferroelectric transitions by symmetry trapping in Cs­(La,Nd)­Nb_2_O_7_,^28^ highlight the importance of investigating structural instabilities in *n* = 2 DJ perovskites.

The complex structural chemistry of RbNdM_2_O_7_ makes this system particularly intriguing for subtle structural analysis, positioning it as a promising candidate for further exploration. In the case of RbNdM_2_O_7_, previous experimental investigations primarily focused on identifying the various polymorphs involved during the structural transformations. However, the transition temperature to the highly symmetric *P*4/*mmm* phase has yet to be determined. Additionally, deeper insights into these compounds, focusing on lattice instabilities, thermal expansion behavior, etc., would be valuable for a more complete understanding of the crystal structure through sequential structural transitions. Such investigations can provide crucial information about the underlying mechanisms driving these transitions and their effect on the overall properties of the materials. Moreover, for energy applications, it has been evidenced that possible excitation processes including the partly occupied 4f states, may play an important ide for efficient photocatalytic activity.^29^

Regarding the computational approach, some results for RbNdM_2_O_7_ have been reported; however, these have been done by employing the conventional (semi)­local functionals of the density. Some works have considered the three 4f electrons of Nd as being frozen in the core,^7,21,22^ while another work carried out by Machida et al.,^29^ employed the all-electron full-potential linear augmented plane-wave (FLAPW) method together with the local density approximation as the exchange-correlation (xc) functional. While, and for the first consideration, the semilocal xc functional (i.e., PBEsol) sufficed for the studied purposes (energetic ordering of phases, spontaneous polarization), the latter consideration impacts the description of the electronic properties, in the sense that these DFT functionals incorrectly describe the strongly correlated states of the f-electrons (and even the *d*-states). To the best of our knowledge, there have been no further attempts to employ the on-site Hubbard corrections (DFT + *U*) on these type of materials, and hence, a renewed effort using such frameworks may shed light on the respective electronic properties.^30^

In this context, along with theoretical calculations, we have conducted an in-depth structural analysis of RbNdTa_2_O_7_ (RNTO) by conducting temperature-dependent X-ray and neutron powder diffraction focusing on the phase transitions, structural distortions, and thermal expansion behavior. To further explore the lattice dynamics, we have measured the temperature-dependent Raman spectra, which, to the best of our knowledge, has not been reported for A′NdM_2_O_7_. Understanding these aspects can provide crucial information about the complex interplay of structural distortions and phase transitions, clarifying the unique properties and potential applications of these type of materials.

## Methodology

### Experimental Section

#### Sample Preparation

Polycrystalline RNTO was prepared following a conventional solid-state ceramic method,^21^ using Rb_2_CO_3_ (99.8%), Nd_2_O_3_ (99.99%), and Ta_2_O_5_ (99.993%). Nd_2_O_3_ and Ta_2_O_5_ were preheated at 900 °C before being used. Initially, stoichiometric amounts of the oxides were ground together using isopropanol as the medium. Eventually 50% excess of Rb_2_CO_3_ (to compensate for the loss of volatile Rb at higher temperatures) was added to the dry mixture, ground again for 10 min and heated at 850 °C in the air for 12 h. The resultant powder is then ground, pelletized, and heated at 1050 °C for 12 h (4 times), with intermediate grindings, using a heating rate of 5 °C min^–1^ and a cooling rate of 10 °C min^–1^. Finally, the sample was washed with distilled water (to remove any remaining Rb oxides), centrifuged, and the residue was dried for 12 h at 140 °C in air. This complete process enabled the preparation of a phase pure sample, which was used for further characterizations.

#### Characterization

The progress of sample formation between the synthesis steps and the final purity of the sample was assessed by monitoring the powder X-ray diraction (PXRD) data collected using a Rigaku SmartLab X-ray diffractometer (45 kV, 200 mA) operating with Cu Kα in Bragg–Brentano geometry. Variable-temperature PXRD data from 300 to 1250 K was collected using a high-temperature Anton Paar HKT1200 furnace, on a Bruker d8 series 2 diffractometer with a Lynxeye detector and Cu Kα_1/2_ at Durham University, U.K. Temperature calibration was performed using an Al_2_O_3_ external standard. The sequential profile fitting of the PXRD patterns was carried out using Topas.^31^ Time-of-flight (TOF) neutron powder diffraction (NPD) patterns were collected with the POWGEN^32^ instrument at the Oak Ridge National Laboratory, Tennessee. A central wavelength of 1.5 Å was used, covering a *d*-spacing of 0.5–10.84 Å. Approximately 1.8 g of powder sample was loaded in a cylindrical vanadium can with a diameter of 6 mm. The MICAS vacuum furnace was used. Variable-temperature diffraction data were collected at room temperature, 600, 800, 970, 1000, 1100, 1150, 1200, and 1275 K for 1–2 h. A heating rate of 5 °C/min was used. The crystal structure analysis of the NPD patterns by Rietveld refinements was performed using Fullprof software.^33,34^ The bond lengths, angles, distortions, and bond valence sum of the crystal structures were calculated using BondStr, a tool included in FullProf. The visualization of the crystallographic structures was done with VESTA.^35^ Temperature-dependent Raman spectra measurements were performed from 300 to 800 K to probe the lattice dynamics in detail around the first structural transition. The Raman spectra were recorded using a Renishaw inVia Qontor confocal Raman spectrometer combined with a Linkam heating stage (Model: HFS600E-PB4) and a Peltier-cooled charge-coupled device (CCD) detector with a 633 nm linearly polarized line of a He–Ne laser for excitation. The spectra were recorded in a backscattering geometry, using a 50× objective lens.

### Density Functional Theory

The Vienna *Ab initio* Simulation Package (VASP)^36^ was used within the projector augmented-wave (PAW) scheme. The data sets included nine valence electrons for Rb­[4s^2^4p^6^5s^1^], fourteen valence electrons for Nd­[5s^2^5p^6^5d^1^4f^3^6s^2^], eleven valence electrons for Ta­[5p^6^6s^2^5d^3^] and six valence electrons for O­[2s^2^2p^4^]. Total energy convergence was achieved with a plane-wave kinetic-energy cut-off of 700 eV. In the present theoretical study, the spin-polarized DFT + *U* method was employed with the generalized-gradient approximation (GGA) with the Perdew, Burke, and Ernzerhof (PBE) parametrization applied as the exchange-correlation functional^37,38^ and a Hubbard on-site potential proposed by Dudarev et al.^39^ Within the DFT + *U* method, the potential energy is supplemented with a Hubbard-like term to treat the strongly correlated f-states of Nd. The *U* value was obtained by comparing the partial density of states (PDOS) between all of the studied polymorphs. Since our purpose is to compare the energetic trend between the structural phases, we employed the *U*_eff_ = 6 eV for all systems since we observed that the occupied Nd–f states were correctly treated as being localized and must below the Fermi Energy, with the VBM being mainly composed of O p-states, as to what occurs in other oxides.

The sampling of the Brillouin-zone (BZ) was converged with a Γ-centered Monkhorst–Pack^40^ grid employing different mesh densities according to the primitive-cell of the five different studied structures: *P*4/*mmm* (S.G. 123) → 12 × 12 × 6; *I*4/*mcm* (S.G. 140) → 6 × 6 × 6; *Cmce* (S.G. 64) → 10 × 10 × 2; *I2cm* (S.G. 46) → 6 × 6 × 6; *Pc2*_1_ *n* (S.G. 33) → 2 × 2 × 8.

Calculations were performed by considering high-spin densities, which therefore resulted in ferromagnetic ordering. The antiferromagnetic states have also been computed for the sake of comparison and for the ground-state systems. Since the energy difference between both magnetic orderings is similar, we applied throughout the work the ferromagnetic configuration.

## Results and Discussion

### Low-Symmetry Structural Phases as Potential Ground-State Polymorphs

RNTO has been identified as crystallizing into a polar orthorhombic ground state structure with *I*2*cm* space-group symmetry (Figure 1) at room temperature. Upon heating, RNTO exhibits sequential structural phase transitions *I2cm* → *Cmce* (*T*_1_ ∼ 500 K) → *I*4/*mcm* (*T*_2_ ∼ 950 K) → *P*4/*mmm* (*T*_3_ not yet characterized).^21^

**Figure 1:**
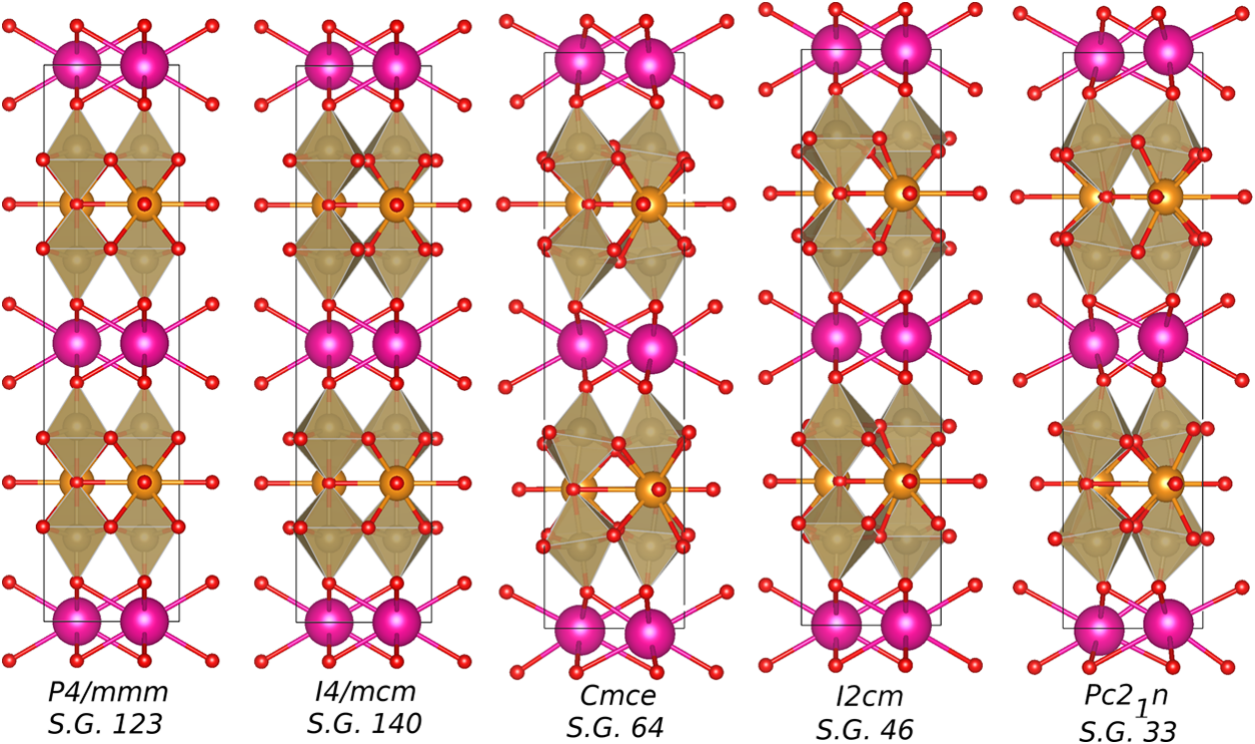
Five studied polymorphs of RNTO. In all images, the pink spheres represent the Rb ions, the orange spheres are the Nd ions, and the brown polyhedra are the TaO_6_ octahedra with the O atom (red).

To confirm the transition pathway by DFT calculations, we have computed the heats of formation to assess the relative enthalpic stabilities of these four phases along with a hypothetical *Pc*2_1_ *n* polymorph, with respect to the constituent elements, by applying

1 $$\Delta H_{f}=E_{\mathrm{tot}}^{\mathrm{RNTO}}-\left[E_{\mathrm{tot}/\mathrm{atom}}^{\mathrm{Rb}}+E_{\mathrm{tot}/\mathrm{atom}}^{\mathrm{Nd}}+2\times E_{\mathrm{tot}/\mathrm{atom}}^{\mathrm{Ta}}+7\times E_{\mathrm{tot}/\mathrm{atom}}^{O}\right]$$

where *E*_tot_^RNTO^ is the total energy (per formula unit) of the different polymorphs of RNTO, and *E*_tot/atom_^Rb,Nd,Ta,O^ is the total energy per atom of the pure components in their standard states. The calculated heats of formation are tabulated in Table 1, and structures are drawn in Figure 1; we note that the energetic trend is consistent with what is stated in refs 21 and 22. We observe from Table 1, that the two lowest symmetry structures, *I*2*cm* and *Pc*2_1_ *n*, are essentially equienergetic; therefore, there could be a possibility for the two phases to coexist at lower temperatures or under another external perturbation (e.g., pressure).

**Table 1:** Heats of Formation of the Five Polymorphs of RNTO, Calculated from the 0 K Equilibrium Structures, Considering *U*_eff_ = 6 eV^a^

Phase	Δ*H*_f_
*P*4/*mmm*	–32.0816
*I*4/*mcm*	–32.1944
*Cmce*	–32.2724
*I*2*cm*	–32.3119
*Pc*2_1_ *n*	–32.3146

a Values are given in eV per formula unit.

We have also calculated the energetics of the *Pc*2_1_ *n* structure because group-subgroup relations show that a structural transition from *I*2*cm* towards a lower symmetry polymorph, *Pna*2_1_, is possible (see Figure 2). *Pna*2_1_ is an alternate settings of the space-group *Pc*2_1_ *n*. Moreover, the *Cmce* phase can also be lowered toward *Pna*2_1_, through the possible pathway of three orthorhombic structures: *Aba*2, *Pnma* or *Pccn*. The group-subgroup diagram shows that no continuous structural phase transition can occur between the space groups *Cmce* and *I*2*cm*. Such a transition would be a discontinuous or first-order phase transition.

**Figure 2:**
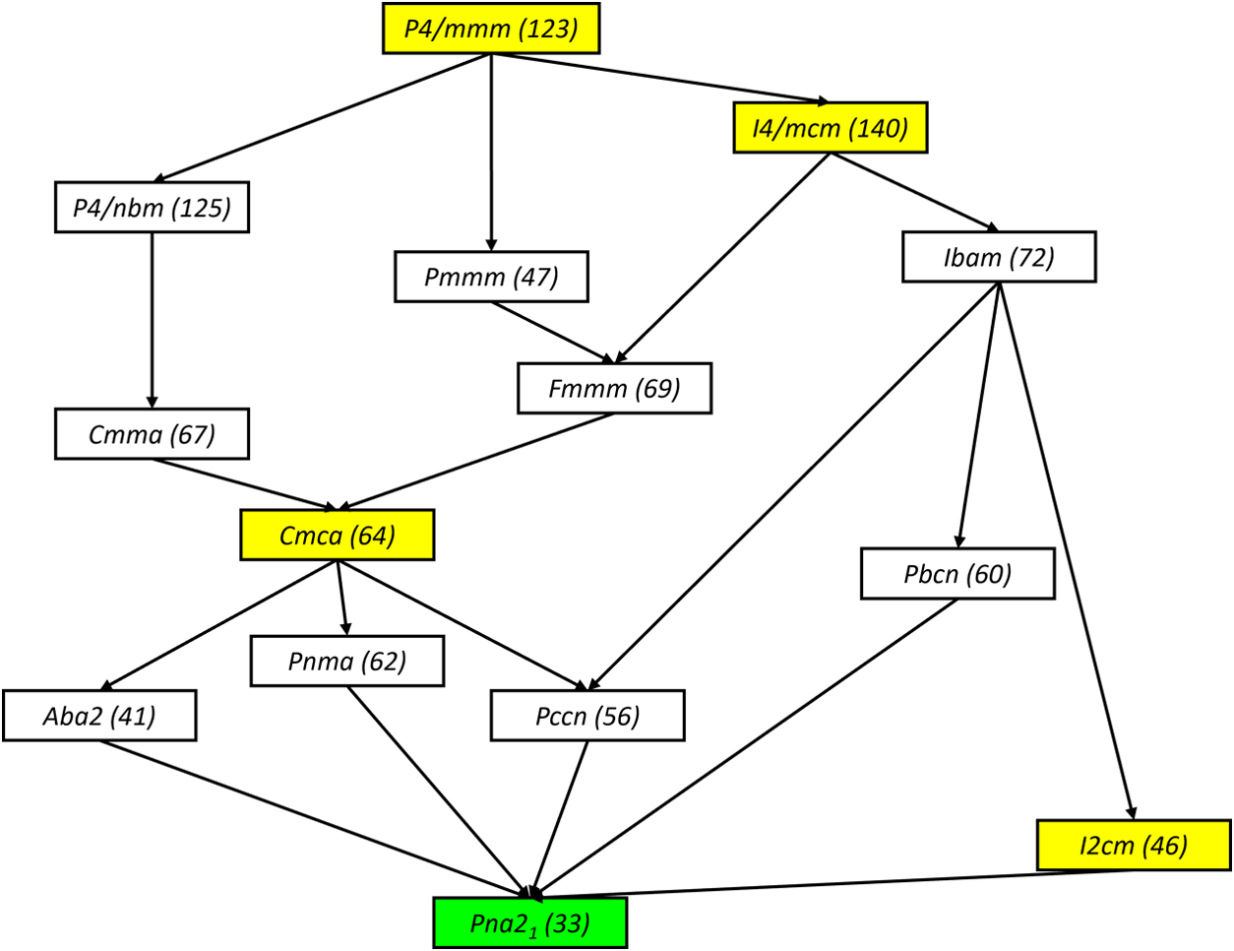
Group-subgroup relation obtained using Isodistort^41^­(*H.T. Stokes, D.M. Hatch, and B.J. Campbell, ISOTROPY Software Suite, iso.byu.edu.*) The yellow box represents the previously discovered structures.^21^ The green box represents the *Pna*2_1_ symmetry, which is energetically similar to *I*2*cm*.

### Room Temperature Structural Study

A preliminary analysis of laboratory PXRD collected at room temperature from the RNTO confirmed a high-quality polycrystalline sample. Structural refinement was conducted using NPD data collected at 300 K, using two models: (i) *I*2*cm*, based on a previous report,^21^ and (ii) *Pc*2_1_ *n*, identified from our phase stability analysis (above section). The resulting Rietveld fits are depicted in Figure 3, and the corresponding structural parameters are provided as Supporting Information (Table S1).

**Figure 3:**
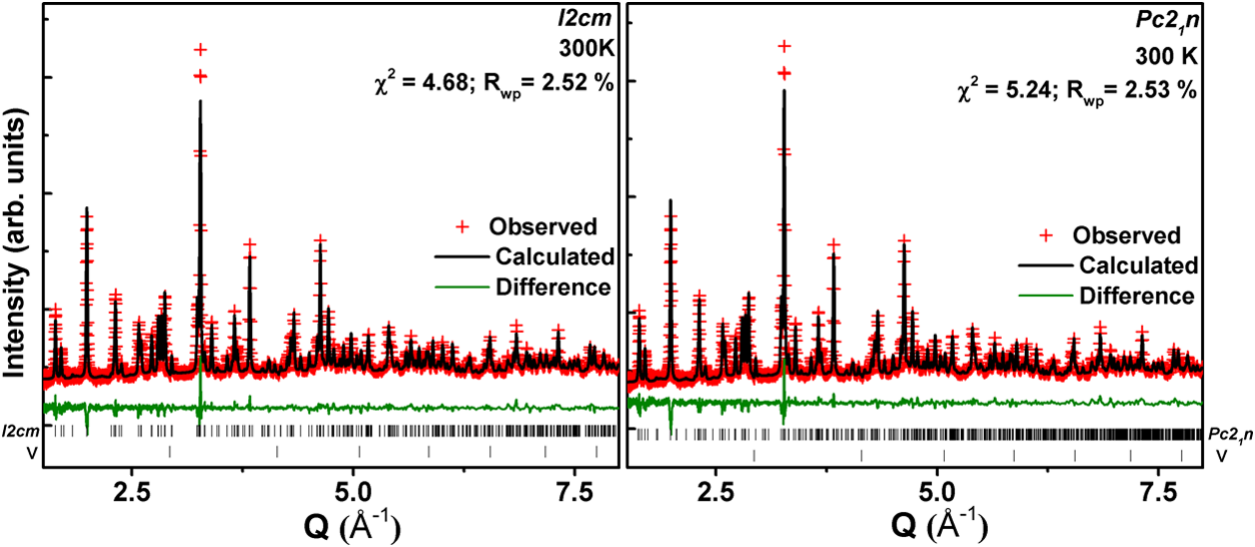
Structural refinement plots of RNTO at 300 K from NPD data (*Q* = 2π/*d*).

Refinements with both models successfully converged after refining the scale factor, lattice parameters, peak shape parameters, atomic coordinates, and isotropic thermal displacement parameter. The observed diffraction peaks adhere to the reflection conditions from the *I*2*cm* model while the extra peaks allowed in *Pc*2_1_ *n* such as (102), (104), (111), etc., where *hkl*: *h* + *k* + *l* = odd, are not evident in the diffraction pattern. Thus, the NPD analysis does not provide any evidence for the *Pc*2_1_ *n* structure at room temperature in the investigated sample. Nevertheless, it is important to note that we cannot rule out the possibility of RNTO crystallizing in *Pc*2_1_ *n* (Table 1), indicating the potential for stabilizing the *Pc*2_1_ *n* phase at lower temperature, or pressure.

### Variable-Temperature PXRD: Cell Parameters and Thermal Expansion

To accurately track the temperature evolution of the cell parameters, PXRD patterns were collected from 316 to 1250 K in 20 K increments, followed by cooling to 290 K, resulting in a total of 98 patterns (Figure 4). It is evident from Figure 4 that an additional peak emerges above 1163 K, with 2θ = 28.50°, identified as orthorhombic NdTaO_4_. Intriguingly, this phase appeared at a lower temperature than the synthesis temperature (1323 K) of RNTO. This suggests that the volatility of Rb ions may contribute to the formation of this phase. (During the synthesis, excess Rb_2_CO_3_ was employed to compensate for potential losses due to volatility. However, excess Rb_2_O was removed after the final annealing.) Even after the emergence of this additional phase, subsequent cooling reveals that the PXRD patterns of RNTO at 290 K predominantly exhibit the *I*2*cm* phase (85–90 %). This observation suggests that the sequence of the structural transitions remains unaffected. Consequently, we proceeded with the analysis of structural changes at varying temperatures using high-temperature PXRD and NPD patterns to explore the phase transitions and determine the unidentified transition temperature to the highest symmetry *P*4/*mmm* phase.

**Figure 4:**
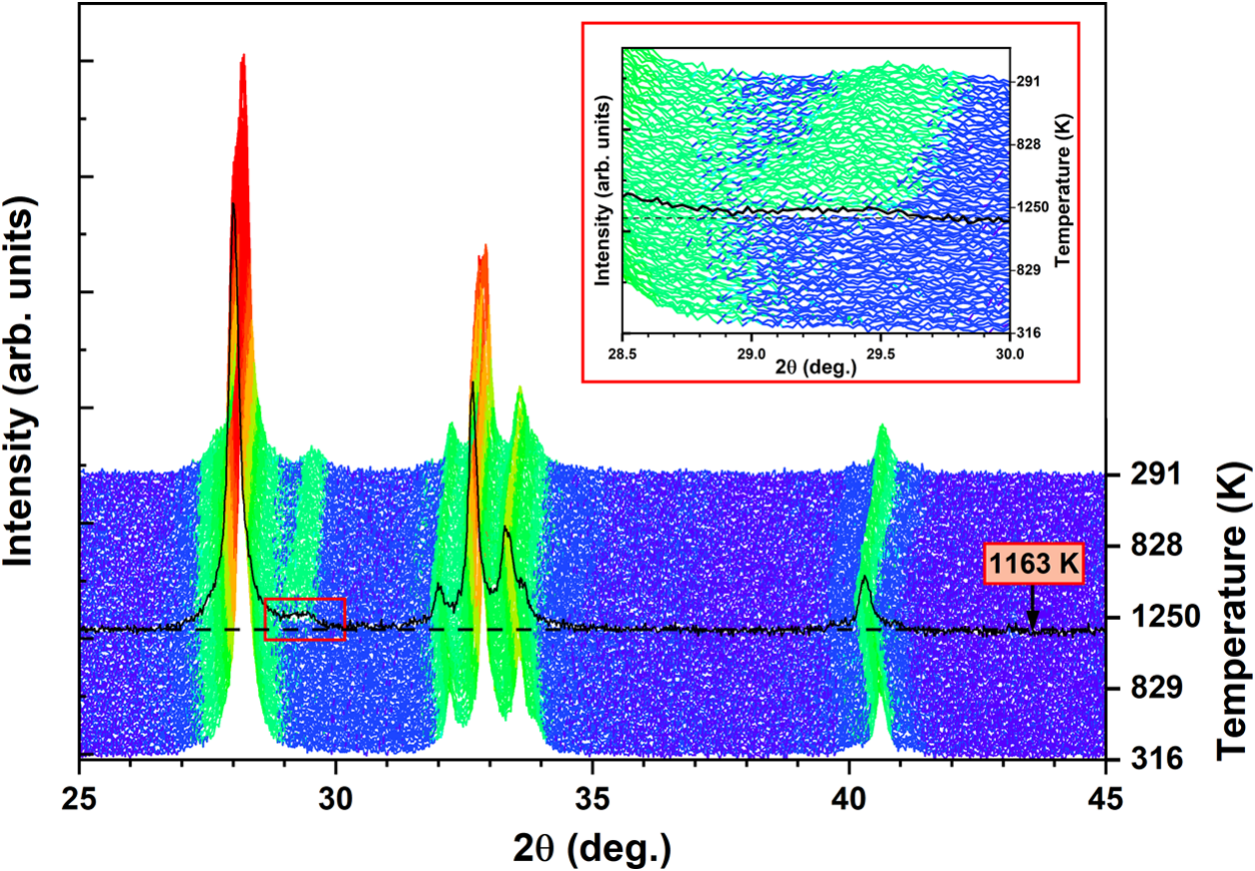
Temperature variation PXRD pattern with the inset showing a portion zoomed in to visualize the onset of the second phase (NdTaO_4_).

All 98 PXRD patterns were profile fitted using the *I*2*cm* model to analyze the temperature evolution of lattice parameters during the structural phase transitions. The temperature evolution of normalized unit cell parameters is shown in Figure 5. As the temperature increases, all of the cell parameters exhibit a continuous increase up to 500 K (*T*_1_). Around *T*_1_, the cell parameters show abrupt changes: (i) an increase in *a*_*P*_ and *b_P_*, (ii) a decrease in *c_P_*, and (iii) the presence of thermal hysteresis. This behavior is consistent with the reported^21^ first-order structural transition of RNTO from *I*2*cm* to *Cmce*.

**Figure 5:**
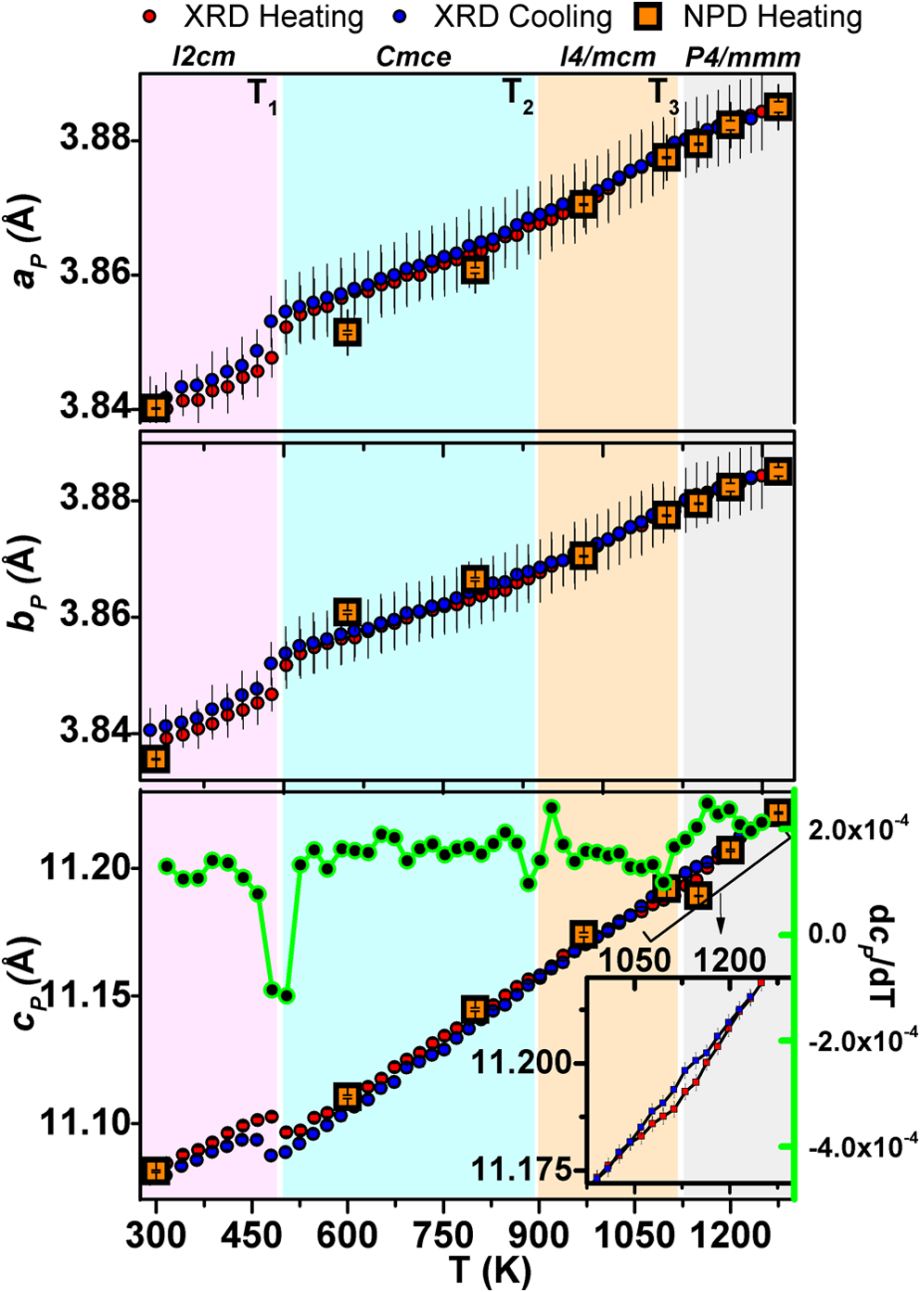
Evolution of normalized cell parameters with temperature, obtained after PXRD and NPD refinement. The plot of *c*_*P*_ includes its temperature derivative (shown in green) and an enlarged view of *c*_*P*_ in the high-temperature region, as the inset. [*a*_*P*_, *b*_*P*_, and *c*_*P*_ represents the normalized cell parameters to the undistorted aristotype symmetry, *P*4/*mmm. I*2*cm*: $a∼b=\sqrt{2}a_{p}$ , *c = 2c*_*P*_; *Cmce*: *a* ∼ *b* = 2*a*_*P*_, *c* = 2*c*_*P*_ and *I*4/*mcm*: $a=b=\sqrt{2}a_{p}$ , *c* = 2*c*_*P*_].

Following *T*_1_, the cell parameters continue to increase steadily. However, a notable change occurs around 900 K (*T*_2_) in the derivative plot of *c*_*P*_ with temperature, supporting the reported^21^ second phase transition in RNTO (occurring at 950 K) from *Cmce* to *I*4/*mcm*. Further analysis reveals a slight change in the derivative plot of *c*_*P*_ around 1100 K (*T*_3_), which could be attributed to the previously unidentified transition temperature from *I*4/*mcm* to *P*4/*mmm*. An enlarged view of the *c*_*P*_ variation in the high-temperature region, depicted in the inset of Figure 5, may indicate a small thermal hysteresis around *T*_3_.

Figure 6 shows the temperature evolution of the relative change in lattice parameters and volume [Δ*L*/*L*_0_ and (Δ*V*/*V*_0_)^1/3^]. The *a*/*b* lattice parameters and volume display positive thermal expansion (PTE) throughout the measured temperature range while the *c* exhibits NTE across the first-order phase transition at *T*_1_. Also, the phase transition at *T*_1_ is reversible and shows thermal hysteresis. Thus, on heating/cooling, the RNTO undergoes a rapid phase transition (*I*2*cm* → *Cmce*), over an interval of ∼50 K, that is accompanied by a large and anisotropic change in the unit cell parameters with relatively small volume change. This is similar to what has been reported for thermosalient compounds, although in our case, we observe the NTE within a small temperature range.^42^ The inset of Figure 6 shows the coefficient of thermal expansion (CTE) across the *T*_1_. The obtained CTE values are comparable to those reported for other DJ oxides.^43^

**Figure 6:**
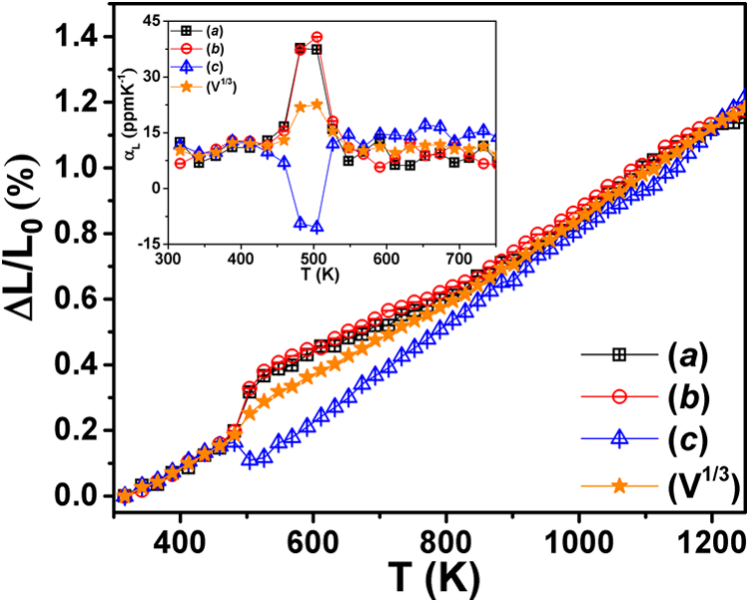
Relative change in lattice parameters and volume with temperature. Inset: Variation of thermal expansion coefficients with temperature.

Utilizing PASCal,^44,45^ we computed the thermal expansion indicatrices, and the three-dimensional representations are shown in Figure 7. Within this indicatrix, the distance between the surface and the origin in a given direction *r* corresponds to the magnitude of CTE (α) in that direction, including negative values as needed. These show close to isotropic PTE in the *I2cm* phase, while all other phases exhibit anisotropic PTE. In the *P*4/*mmm* phase (*a* ^0^a^0^c^0^), the observed expansion along the *c-*axis is consistent with typical behavior in layered perovskites with tetragonal symmetry.^43^ The distinct thermal expansion behavior of RNTO across the different crystallographic phases likely arises from the phase-specific constraints on atomic movements/flexibility due to characteristic tilts/distortions.

**Figure 7:**
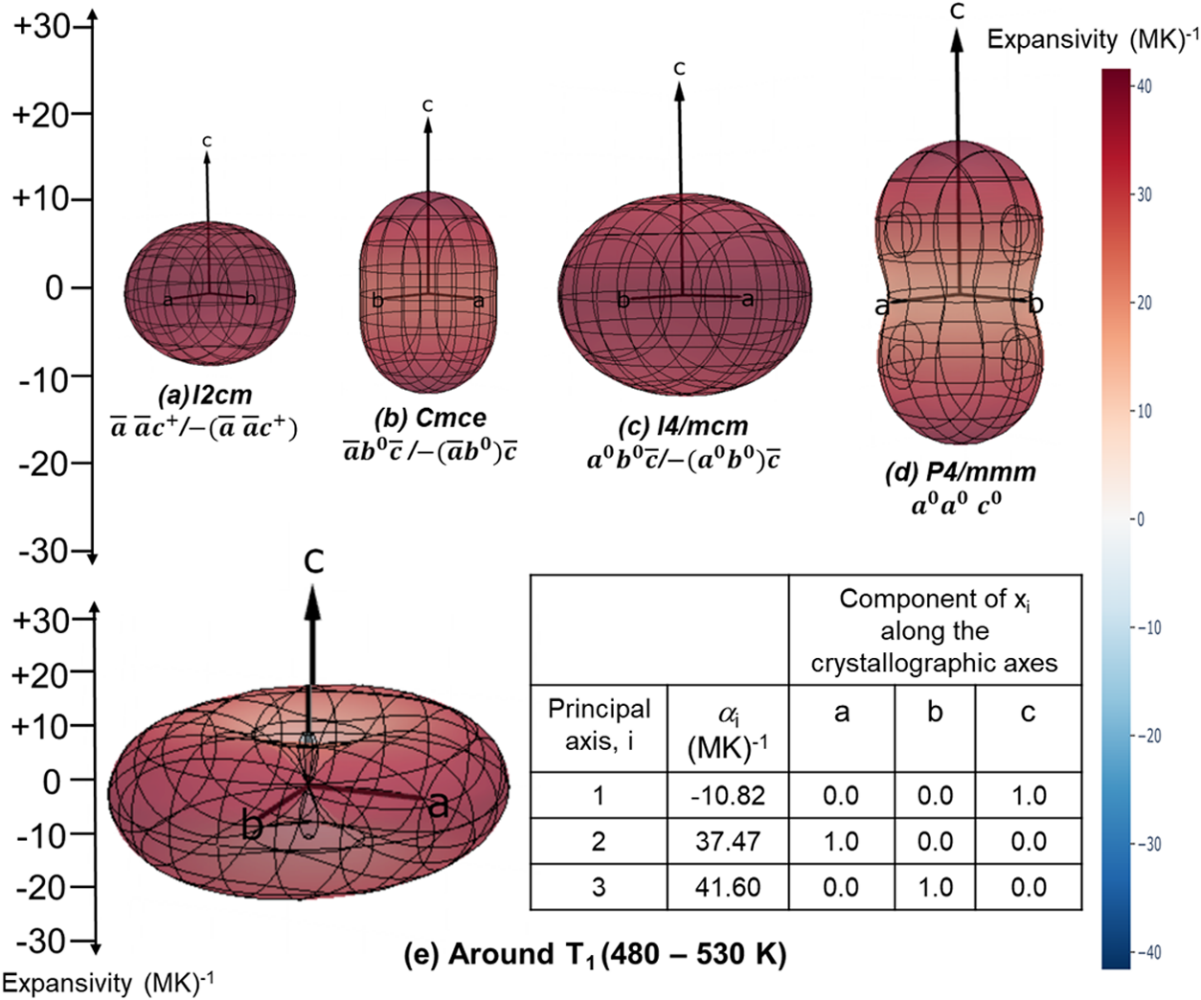
(a–d) Thermal expansion indicatrices for different structural phases obtained from PASCal;^45^ (e) thermal expansion indicatrix around *T*_1_, along with the table containing principal CTE and corresponding principal axes.

Across the first-order transition (*T*_1_) from 480 to 530 K, the expansivity indicatrix shows compression along the *c-*direction with a negative CTE (α_1_ = −11 ppmK^–1^). However, the positive CTEs along the *a*/*b* directions (α_2_ = 38 ppmK^–1^ and α_1_ = 42 ppmK^–1^) result in positive volume thermal expansion (α_*V*_ = 68 ppm K^–1^). Outside this range, the RNTO shows PTE in all directions. Thus, RNTO exhibits uniaxial NTE along the *c* direction across the first-order transition *T*_1_, while the volume CTE remains positive, similar to other layered perovskites.^46–48^ Within this narrow temperature range (480–530 K), the negative CTE observed of RNTO, α_1_ = −11 ppmK^–1^, is comparable to that of commercial NTE materials currently in use, i.e., ZrW_2_O_8_ with −9 ppmK^–1^.^49^ The origin of the observed uniaxial NTE in RNTO across *T*_1_ will be explored in a later section utilizing the DFT results.

### Variable-Temperature NPD: Phase Transitions

To track the previously unidentified structural transition temperature to the high-symmetry phase *P*4/*mmm*, we conducted variable-temperature NPD, with a particular focus on the high-temperature region (around *T*_3_). Rietveld refinements of crystal structures using NPD patterns over the measured temperature range were performed using different structural phases: (i) the *Cmce* model for 600 and 800 K, (ii) the *I*4/*mcm* model for 970, 1000, and 1100 K, and (iii) the *P*4/*mmm* model for 1150, 1200, and 1275 K. The refinements at higher temperatures improved significantly after including an additional phase NdTaO_4_ from 970 K onward (2.6% at 970 K which reaches 3.7% at 1250 K). Figure 8 (Left) displays the observed, calculated, and difference plots of the NPD fit at selected temperatures. The magnified view of the Q-range from 2.5–2.9 Å^–1^ in the measured temperature range, shown in Figure 8 (right), clearly demonstrates the changes in diffraction patterns corresponding to the different structural phases and confirm *T*_3_ lies between 1100 and 1150 K. Temperature evolution of cell parameters obtained from NPD have been included in Figure 5. NPD refinement at 1100 K shows plausible coexistence of *I*4/*mcm* and *P*4/*mmm* phases which corroborates the thermal hysteresis observed at higher temperature in the derivative plot of *c*_*P*_, shown in the inset of Figure 5. The structural parameters obtained after Rietveld refinement of NPD at different temperatures are given as Supporting Information (Tables S2–S4).

**Figure 8:**
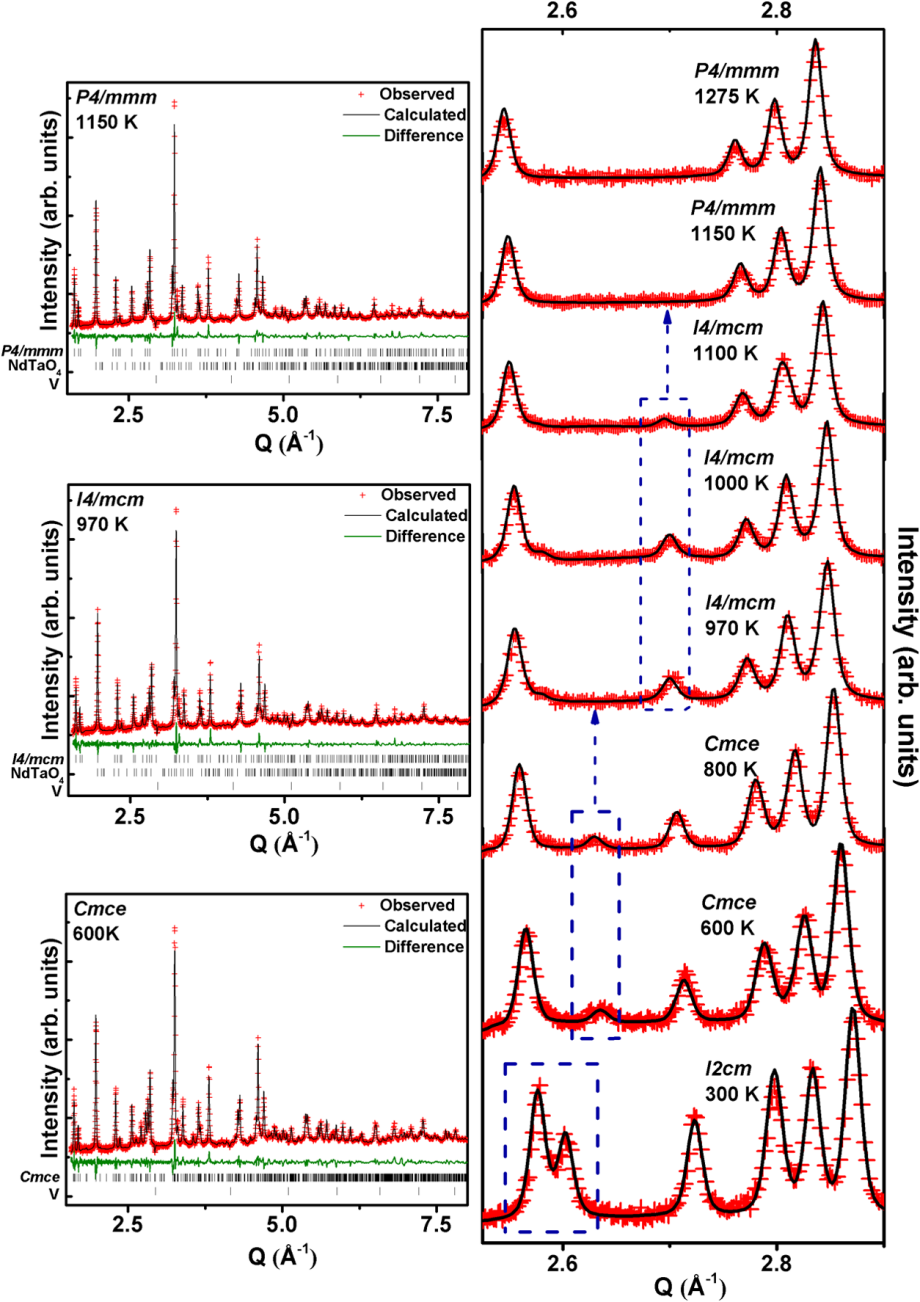
Left panel: Observed, calculated, and difference plots from the structure refinement of RNTO using NPD data collected at 600, 970, and 1150 K. Tick marks correspond to the phases used in the refinement. Right panel: Stacked plots of the magnified Q-range (2.5–2.9 Å^–1^) from 300 to 1275 K, highlighting peak evolution during the sequential structural phase transition (*Q* = 2π/*d*).

### Bond Lengths and Polyhedral Distortions

In RNTO, the structural modifications at phase transitions primarily impact the TaO_6_ octahedral bilayer. Therefore, we depict the evolving structural changes associated with the octahedral bilayer, obtained from the structural refinement of NPD, in Figure 9. The TaO_6_ octahedral tilt is clearly visible in low-symmetry structures such as *I2cm* and *Cmce*. A significant difference in bond distances is observed between Ta and the two axial oxygens (Ta–O_ax_): (i) the longer axial bond, shared between the octahedra, represented as “Ta–O_ax–L_” and (ii) the shorter axial bond, pointing outward from the octahedra, represented as “Ta–O_ax–S_”. Ta–O_ax–S_ is shared between Rb–O and the perovskite-like NdTa_2_O_6_ layer (Figure 1). It is notable that the compound exhibits both short and long Ta–O_ax_ bonds in all the phases, which confirms that the Ta ions consistently exhibit displacement toward the Rb–O layers, attributed to the SOJT effect of Ta^5+^ (d^0^).^50^

**Figure 9:**
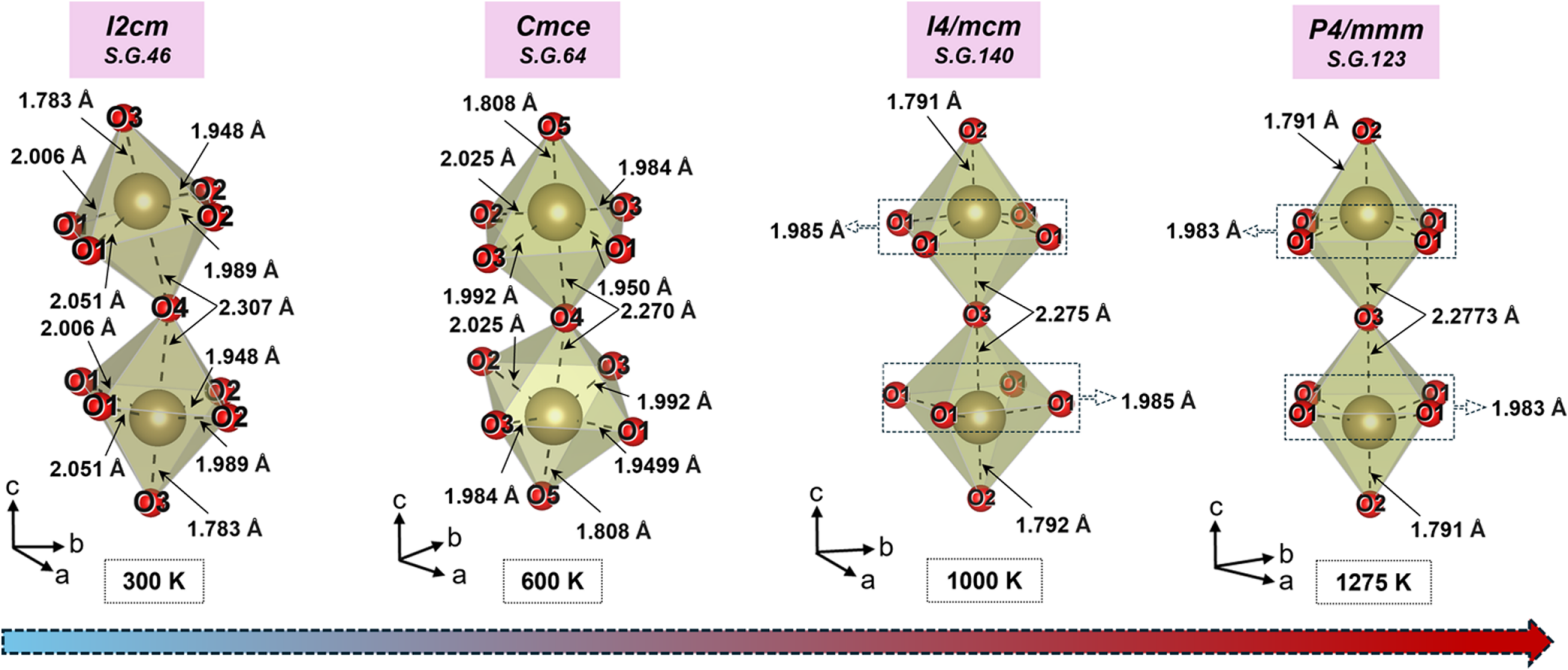
Comparison of octahedral (TaO_6_) equatorial and axial bond lengths in different structural phases.

The bond lengths obtained at room temperature for Rb–O, Nd–O and Ta–O are consistent with those reported by Zhu et al.^22^ With increasing temperature, the average equatorial bond length of the octahedra, Ta–O_eq_, decreases from 1.999(1) Å at 300 K (*I2cm*) to 1.988(1) Å at 600 K (*Cmce*). Similarly, a notable reduction is observed in the Ta–O_ax–L_ bond length, which decreases from 2.307(1) Å at 300 K to 2.270(1) Å at 600 K. At the same time, the Ta–O_ax–S_ bond length is increased from 1.783(1) Å at 300 K to 1.808(1) Å at 600 K. The simultaneous reduction of Ta–O_eq_ and Ta–O_ax–L_ bond lengths, alongside the increase in Ta–O_ax–S_ at 600 K, suggests the movement of Ta ions toward the O_eq_ plane, indicating reduction in Ta-off-centering/SOJT effect in the *Cmce* symmetry. In the highest symmetry structure *P*4/*mmm*, the Ta–O_ax–L_ bond length slightly increases, from 2.270(1) Å [600 *K*] to 2.277(1) Å [1275 K], while the Ta–O_ax–S_ bond length reduces from 1.808(1) to 1.791(1) Å, correspondingly. These structural changes suggest varying influences on the balance of SOJT and other effects throughout the transitions, with Ta remaining off-centered even at 1275 K.

Figure 10 shows the temperature dependence of distortion index of bond lengths for Rb, Nd and Ta polyhedra (where distortion index, $D=\frac{1}{n}\sum_{i=1}^{n}\frac{\left|l_{i}-l_{\mathrm{av}}\right|}{l_{\mathrm{av}}}$ , with *l*_*i*_ the distance from the central atom to the *i* ^th^ coordinating atom, and *l*_av_ the average bond length). The variation in average bond lengths with temperature is shown in the respective insets. In the *I2cm* structure, Nd polyhedra exhibit the highest distortion (12 × 10^–3^ Å), followed by Ta octahedra (6 × 10^–3^ Å) while Rb polyhedra display the lowest distortion (3 × 10^–3^ Å). The polyhedral distortion of Rb, located between the perovskite layers, remains relatively consistent in the orthorhombic phases (*I2cm* and *Cmce*) and becomes zero in the tetragonal phases (*I*4/*mcm* and *P*4/*mmm*), while the average Rb–O bond length increases with the temperature. Within the perovskite layer, Nd polyhedra show a systematic reduction in distortion with increasing temperature, becoming undistorted at 1100 K, whereas the Ta octahedral distortion decreases from the *I2cm* to *Cmce* phase and thereafter shows no significant change with increasing temperature. Furthermore, with rising temperature, the average Nd–O and Ta–O bond lengths exhibit a more pronounced reduction from the *I2cm* to *Cmce* phase, indicating compression confined to the perovskite layer across the first-order transition. This behavior reflects the observed uniaxial NTE discussed in the section of thermal expansion (Figure 7(e)), and will be further discussed in a later section.

**Figure 10:**
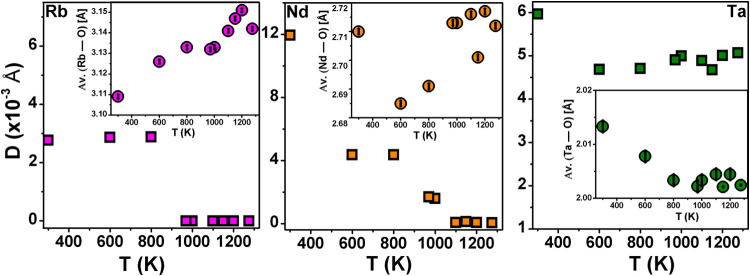
Temperature evolution of the polyhedral distortion index. Insets average bond length variation with temperature (ideal bond lenghts: Rb–O = 3.0324 Å, Nd–O = 2.6179 Å, and Ta–O = 1.9875 Å).

Further, we have calculated the degree of the Ta off-center distortion in the octahedron to provide the temperature dependence of the SOJT effect, as shown in Figure 11. The degree of off-centering is estimated as $\overset{3}{\underset{i=1}{\sum }}\frac{\left|S_{i}-L_{i}\right|}{S_{i}+L_{i}|}$ ,^51^ where S_i_ and *L_i_*_L_ represents the short and long Ta–O bond distances, respectively, in one direction. The degree of Ta-off-centering is maximum in the polar phase *I2cm* (∼0.16 Å) and decreases with an increase in temperature. The persistence of Ta-off-centering across all phases confirms the presence of the SOJT effect throughout the measured temperature range. The mirror symmetry perpendicular to the *c*-axis, however, prevents any net polarization along this axis, rendering the RNTO polar only in the *I2cm* phase.

**Figure 11:**
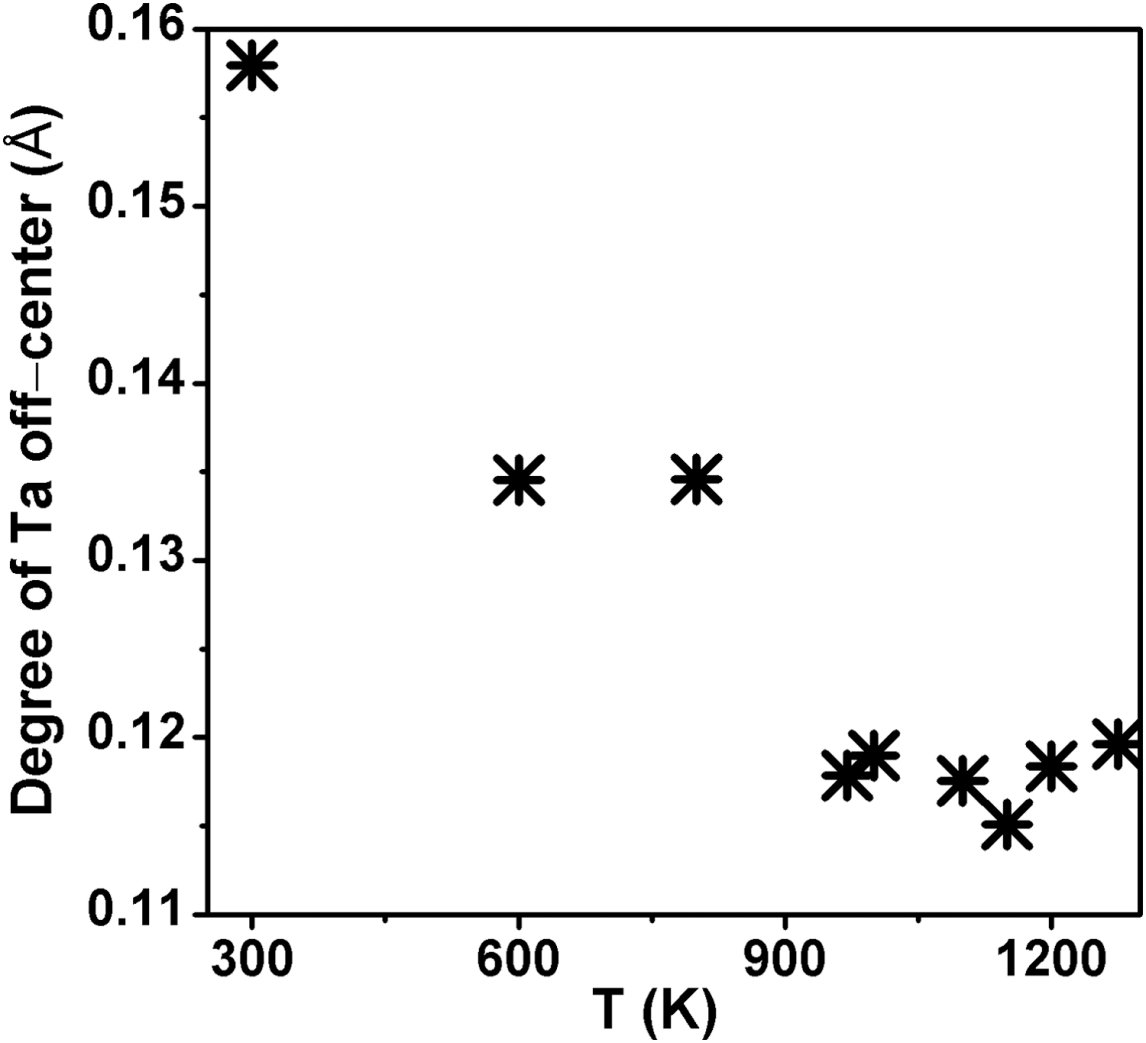
Temperature evolution of the Ta-off-centering.

### Raman Spectral Analysis Across the First Phase Transition

Further, we evaluated the first-order phase transition (*T*_1_) and the related lattice dynamics of RNTO by probing the variable-temperature Raman spectra. The temperature evolution of the Raman spectra exhibits abrupt changes around *T*_1_, consistent with the expected behavior for a first-order structural phase transition. Split bands are observed in *I2cm* compared to *Cmce*, consistent with the increased distortions and lower symmetry.^26,52^

RNTO crystallizes in *I2cm* at room temperature, with 44 ions in the unit cell, implying a total of 66 modes at the zone-center [For Glazer systems with nonprimitive lattices: 3*n*/2 = (3 × 44)/2].^53^ However, the *Cmce* phase can exhibit up to 132 modes [88 ions in the unit cell: 3*n*/2 = (3 × 88) /2]. By consulting the Symmetry-Adapted Modes from the Bilbao Crystallographic Server^54^ and utilizing the crystallographic data obtained in this study from the NPD analysis, the Raman-active symmetry-adapted modes are determined. Out of the total 66 modes present in *I2cm*, 63 are Raman-active, whereas in *Cmce*, only 66 out of the total 132 modes are Raman-active.

The total irreducible representations for Raman-active modes in *I2cm* and *Cmce* are Γ_*R*(*I2cm*)_ = 16A_1_ (*xx*, *yy*, *zz*) + 15A_2_ (*xy*) + 15B_1_ (*xz*) + 17B_2_ (*yz*) and Γ_*R*(*Cmce*)_ = 18A_g_ (*xx*, *yy*, *zz*) + 15B_1g_ (*xy*) + 15B_2g_ (*xz*) + 18B_3g_ (*yz*).

Figure 12 (Bottom) displays the temperature-dependent Raman spectra acquired for RNTO from 100 to 1000 cm^–1^ in the temperature range 300 to 800 K (includes the first phase transition at *T*_1_). The peak positions are identified based on fitting the spectra using a Lorentzian peak shape. (The deconvoluted spectrum at 300 K is provided as Supporting Information, Figure S1). The number of Raman bands observed experimentally for *I2cm* (23) is less than the predicted 63 Raman-active modes. Plausible reasons for this include (i) a limited measured frequency range, (ii) accidental degeneracy of band frequencies, or (iii) low polarizability of several phonons bands resulting in low intensity.^55^

**Figure 12:**
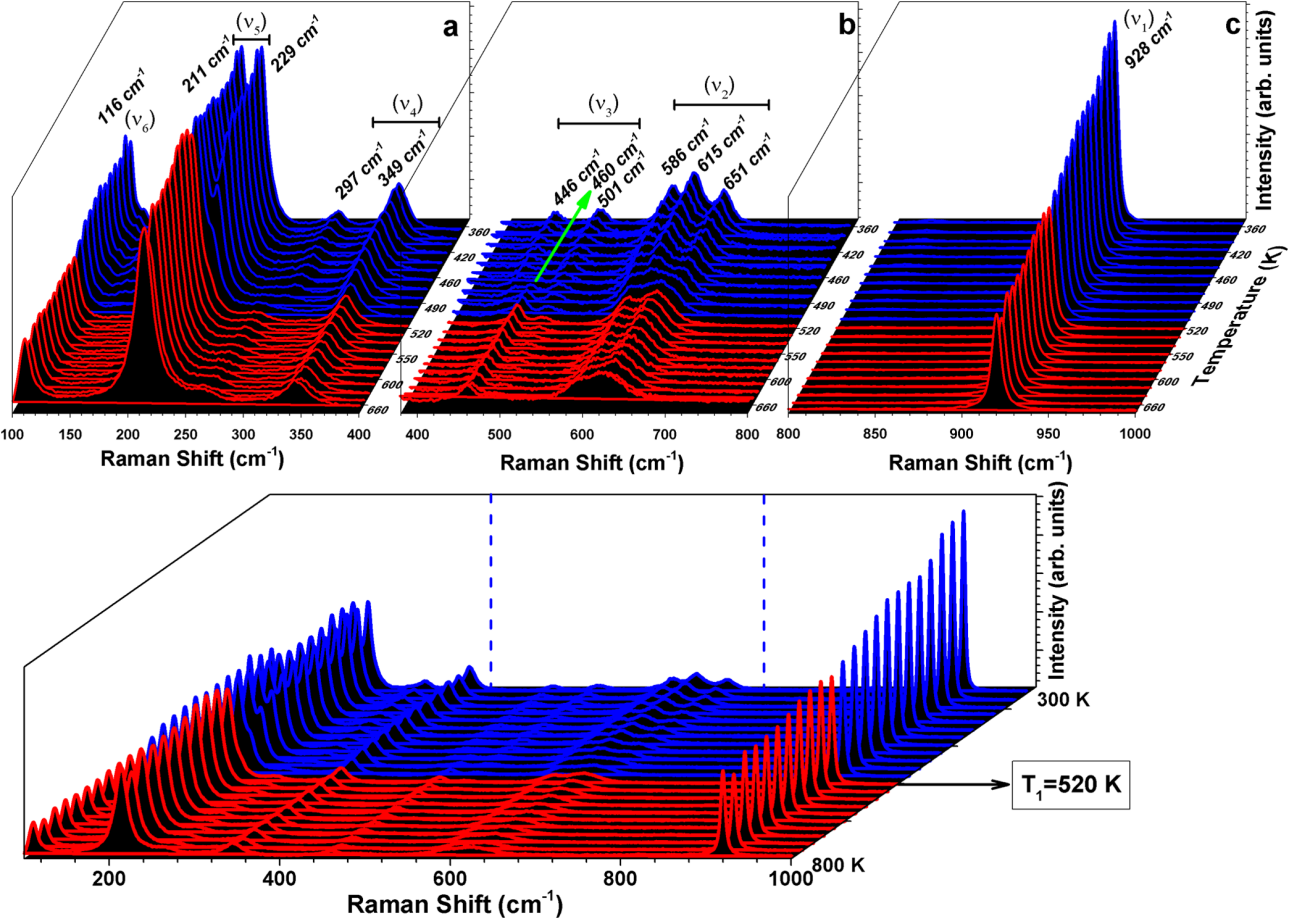
Bottom panel: Temperature-dependent Raman spectra across the first-order phase transition at 520 K, showing the low-temperature *I*2*c*m phase (blue) and high-temperature *Cmce* phase (red). The spectrum is divided into three regions, marked by blue dotted lines. Top panel: Enlarged view of the corresponding spectral regions: (a) 100 to 400 cm^–1^, (b) 400 to 800 cm^–1^, and (c) 800 to 1000 cm^–1^. Significant spectral changes across the transition are noticeable in plots (a) and (b).

Comparison with the Raman spectra of analogous layered perovskites^26,27,56–58^ allows us to assign the higher frequency range, from 400 to 1000 cm^–1^, to TaO_6_ octahedral vibrational modes and the lower frequency range, below 400 cm^–1^, to vibrational modes originating from the Rb–O layer, Nd–O layer, and displacements of Ta ions.

To improve clarity and enhance the visibility of spectral changes, we divided the spectra into three distinct regions, as marked in Figure 12, with the corresponding enlarged plots shown at the top. The prominent and well-defined peak observed in Figure 12(c) at approximately 928 cm^–1^ (ν_1_) can be attributed to the symmetric stretching mode of the TaO_6_ octahedra.^57^ This high frequency is consistent with vibration of the shortest bond (1.7830 Å) in the octahedra (refer Figure 9). Peaks in Figure 12(b) are associated with octahedral asymmetric stretching (590 to 651 cm^–1^: ν_2_) and bending (446 to 581 cm^–1^: ν_3_) vibrations.^52,56–58^ In comparing Figure 12(b,c), the ν_1_ peak shows no significant visual changes during the structural transformation from *I2cm* to *Cmce*, aside from the shift in peak position to lower frequencies. However, ν_2_ and ν_3_ show notable changes, perhaps related to distortions in the TaO_6_ octahedral bonds during the structural transition.

The spectral peaks observed in the low-frequency region Figure 12(a), particularly in the range of 297 to 340 cm^–1^ (ν_4_) and around 200 cm^–1^ (ν_5_), can be associated with the motion of Nd ion.^59^ The peak at ν_5_ could be attributed to Nd translational vibrations whereas the low intensity peaks at ν_4_ might be the octahedral librations due to the symmetric deformation or bending in the octahedron coupled to the displacement of Nd.^59^ The ν_5_ exhibits peak splitting in the *I2cm* phase, comparable to perovskites with *a* ^–^ *a* ^–^ *c* ^+^ tilt.^59^ The peak at 100 cm^–1^ (ν_6_) could be vibrational modes arising from the Rb–O layers.^57,58^

Figure 13 illustrates the temperature dependence of the Raman peak positions. Around the phase transition from *I2cm* to *Cmce* at *T*_1_, the 928 cm^1^ peak (ν_3_) exhibits a sharp change in the position and an increase in full width at half-maximum (fwhm), both of which display thermal hysteresis. Additionally, discontinuities are observed for peaks at 615 (ν_2_), 651 (ν_2_), 446 (ν_3_), 501 (ν_3_), 297 (ν_4_), and 229 (ν_5_) cm^–1^, alongside the emergence of modes around 460 cm^–1^ (ν_3_) and 631 cm^–1^ (ν_2_). These observations are characteristic of a first-order phase transition. The presence of thermal hysteresis in peak position and fwhm (ν_1_), corroborates the thermal hysteresis observed in the variable-temperature PXRD at *T*_1_, and suggests a possible coexistence of the *I2cm* and *Cmce* phases between 470 and 510 K.

**Figure 13:**
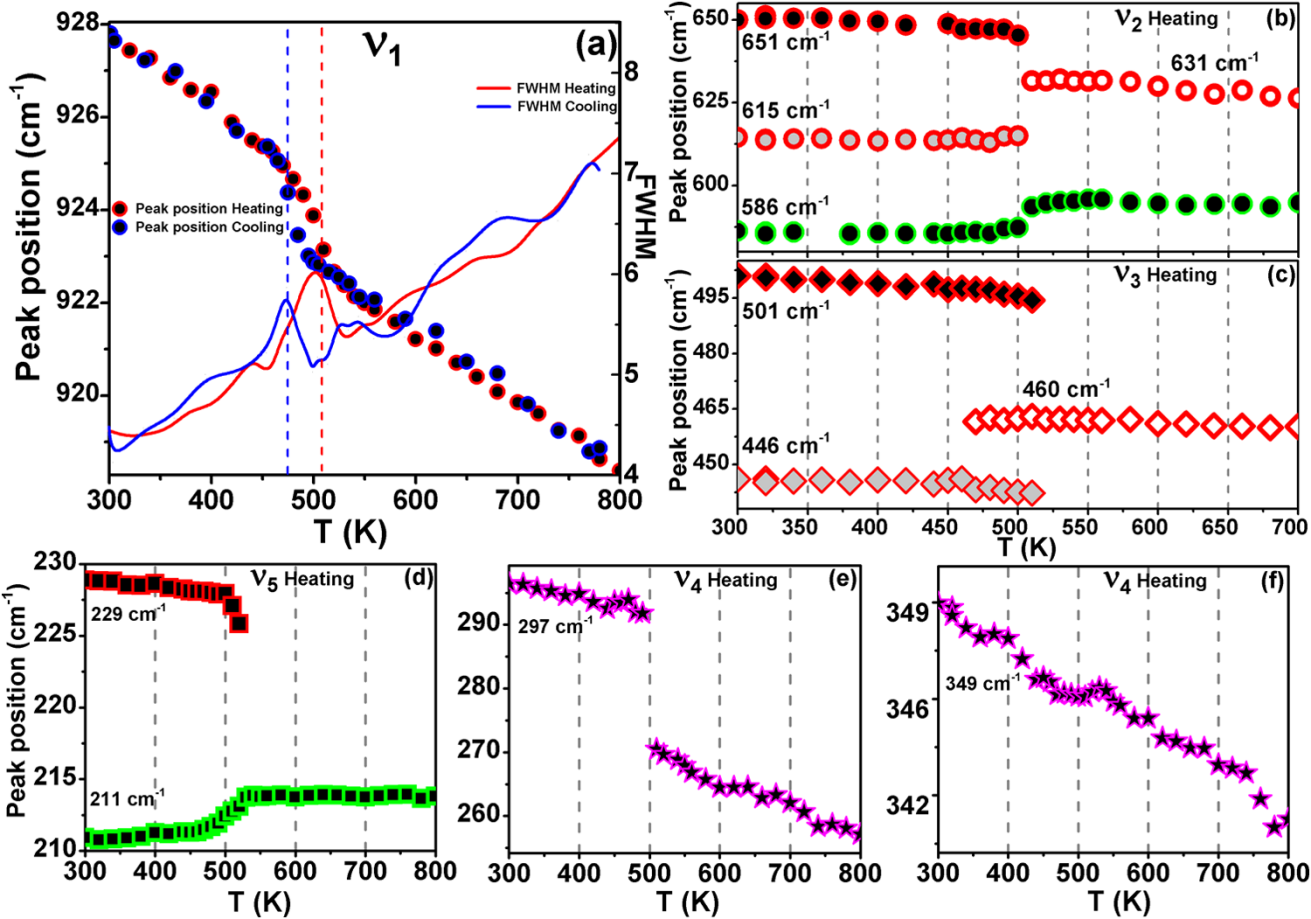
(a) Temperature dependence of peak position and fwhm of the 928 cm^–1^ peak during heating and cooling. (b)–(f) Temperature dependence of Raman peak position during heating for selected Raman peaks: (b) 586, 615, and 651 cm^–1^; (c) 446 and 501 cm^–1^; (d) 211 and 229 cm^–1^; (e) 297 cm^–1^; and (f) 349 cm^–1^.

In temperature-dependent Raman spectra, it is typical to observe peak softening (decrease in frequency of Raman peaks) and broadening as temperature rises. This phenomenon is consistent with the decrease in interatomic interaction (increased bond length) causing peak softening while the increase in phonon population and subsequent decrease in vibrational lifetime (broader distribution of phonon energies and the decreased distinction between individual vibrational modes), in turn, leads to spectral peak broadening.^60^ Apart from the normal peak softening, peaks at 211 (ν_5_) and 586 (ν_2_) cm^–1^ exhibit hardening and the peak at 349 cm^–1^ (ν_4_) shows a a small positional shift around 520 K. This suggets that enhanced bond stiffness during the structural transition is reflected in the modes: (i) octahedral assymmetric stretching (586 cm^–1^), associated with the in-plane octahedral vibrations from Ta–O_eq_; (ii) translational motion of Nd (211 cm^–1^); and (iii) the vibrations from octahedral librations (348 cm^–1^).

We have also calculated the total anharmonicity of various modes in the temperature range 300–510 K, employing the expression $\left(\frac{\partial \nu \left(i\right)}{\partial t}\right)/\nu \left(i\right)$ , adopted from Yuan et al.,^61^ as tabulated in Table 2. Notably, the total anharmonicity predominantly displays negative values across most modes. However, bands at 586 (ν_2_) and 211 (ν_5_) cm^–1^ exhibit hardening with increasing temperature, and show positive total anharmonicity reminiscent of observations in materials exhibiting NTE.^61,62^ Thus, our Raman spectra analysis also provides further confirmation for the first-order transition at *T*_1_ and the associated NTE.

**Table 2:** Total Anharmonicity, $\left(\frac{\partial \nu \left(i\right)}{\partial t}\right)/\nu \left(i\right)$ , up to the Phase Transition, Calculated from Figure 13

Phase	Raman band (cm^–1^)	Total Anharmonicity (K^–1^)
	928 (ν_1_)	–2.44 × 10^–5^
		
	651 (ν_2_)	–0.14 × 10^–5^
	614 (ν_2_)	–1.44 × 10^–5^
	586 (ν_2_)	+5.86 × 10^–5^
		
*I2cm*	501 (ν_3_)	–6.24 × 10^–5^
	446 (ν_3_)	–4.03 × 10^–5^
		
	348 (ν_4_)	–4.23 × 10^–5^
	297 (ν_4_)	–0.42 × 10^–5^
		
	229 (ν_5_)	–6.02 × 10^–5^
	211 (ν_5_)	+4.16 × 10^–5^

### First-Order Transition and Uniaxial NTE

In flexible network structures, NTE is often driven by transverse vibrational modes of rigid polyhedra, known as rigid unit modes (RUMs).^63^ Other possible causes of NTE are electronic in origin, arising from mechanisms such as charge transfer, orbital ordering, magnetostriction, electron–phonon coupling, first-order Jahn–Teller distortion, or cation off-centering displacements due to SOJT effects, etc.^64,65^ Layered perovskites are notable for their unconventional uniaxial or biaxial NTE, driven by layering and condensed symmetry-breaking octahedral rotations coupled to soft phonon modes.^46,47,66–69^

To investigate the origins of uniaxial NTE in RNTO, we have compared the DFT-relaxed *I2cm* and *Cmce* structures to assess the relative importance of the three major structural differences between the two phases: (i) changes in the TaO_6_ octahedral distortions, (ii) changes in the TaO_6_ octahedral tilts, and (iii) changes in the A-site environment when switching from a polar (hybrid improper) to nonpolar structure. The two structures are compared graphically in Figure 14. The impact of structural changes on the unit cell parameters are summarized in Table 3 and the influence on Bond Valence Sums (as a measure of local bonding) and mean metal–metal distances are summarized in Table 4. We emphasize that although the DFT + *U* calculations yield lattice parameters that are somewhat larger than experimental (see Supporting Information Table S5 and corresponding text), what we intend to show here is the trend between these structures. All computational parameters, including *U* were kept the same to allow direct comparison of the results.

**Figure 14:**
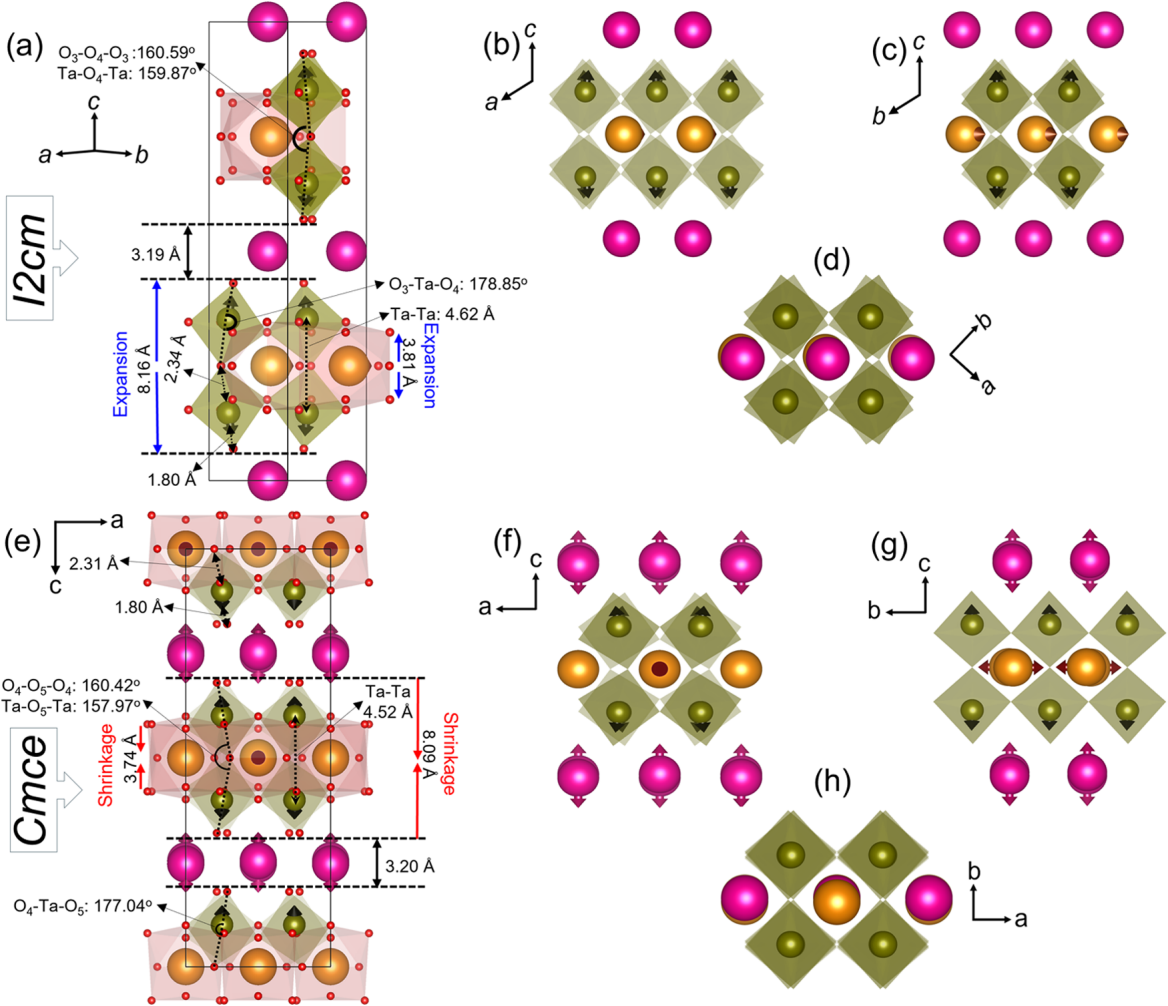
Crystallographic illustrations of the changes in the DFT-relaxed structures for *I2cm* (a–d) and *Cmce* (e–h) [Ta (green), Nd (orange) and Rb (pink)]. Arrows indicate the displacement direction of cations; however, the magnitude of displacement is arbitrary. (a) and (e) Unit cell representation; (b–d, f–h) Representations in different crystallographic planes to visualize the octahedral tilts and displacement direction of the metal ions. Polar displacement of Nd in *I2cm* is visible in (c), while antipolar displacement of Nd in *Cmce* is shown in (g). Octahedral tilting along the *c*-axis is compared in (d) and (h). Nd and Rb cations are shown with the same ionic radii to clearly visualize their displacements. Rb exhibits an antipolar displacement only in *Cmce*.

**Table 3:** DFT+U-Relaxed Structural Analysis I,^a^ Compared with experimental data

	*I2cm*	*Cmce*	relative change (%)
DFT (K)	0	0	
Rb–O layer (Å)	3.187	3.204	+0.53
NdTa_2_O_6_ layer (Å)	8.159	8.085	–0.91
*c*_*P*_ (Å)	11.295	11.240	–0.49
*a*_*P*_ (Å)	3.868	3.900	+0.83
*b*_*P*_ (Å)	3.882	3.910	+0.72
*V* ^*1/3*^ (Å ^3^)	5.535	5.555	+0.36
EXP (K)	316	extrapolated to 316	
*c*_*P*_ (Å)	11.084	11.064	–0.18
*a*_*P*_ (Å)	3.840	3.847	+0.18
*b*_*P*_ (Å)	3.839	3.847	+0.21
*V* ^*1/3*^ (Å ^3^)	5.467	5.471	+0.07

a Layer thickness, lattice parameters (normalized cell parameters to the undistorted aristotype symmetry, *P*4/*mmm*), and relative changes. EXP: Experimental lattice parameter (from Figure 5).

**Table 4:** DFT + *U* -Relaxed Structural Analysis II^a^

	*I2cm*	*Cmce*	relative change %
BVS			
Ta	4.80	4.87	+1.45
Nd	2.75	2.71	–1.45
Rb	0.78	0.73	–6.41
distance (Å)			
⟨Nd–Ta⟩	3.587	3.572	–0.42
⟨Nd–Nd⟩	3.876	3.910	+0.88
⟨Ta–Ta⟩ (equatorial)	3.876	3.903	+0.70
⟨Ta–Ta⟩ (axial)	4.615	4.527	–1.91
⟨Ta–Rb⟩	4.320	4.347	+0.63
⟨Rb–Rb⟩	3.876	3.910	+0.88

a BVS and the distance between the metal ions.

The main difference observed, between the *I2cm* and *Cmce* structures, is a significant reduction in the thickness of the NdTa_2_O_6_ layer (∼−0.90 %) compared to the expansion of the Rb–O layer (∼+0.53 %), resulting in an overall contraction of the unit cell along the *c*–axis of the order of – 0.49 %. This is similar to the magnitude of contraction observed in experimental data for the *c* lattice during the structural transition (∼−0.2 %). A comparison of the experimental lattice parameters is also included in Table 3 (derived from Figure 5) using the experimental value for *I2cm* at 316 K and a 316 K extrapolated value for *Cmce*, which further corroborates the trends observed in the DFT results.

The thickness of the NdTa_2_O_6_ layer is determined by the size of the TaO_6_ octahedra and their tilting angles. As can be seen in Figure 14, the magnitude of the octahedral tilting perpendicular to *c*–axis, defined by the angle between the apical oxygen ions O_ax–S_–O_ax–L_–O_ax–S_ (O_3_–O_4_–O_3_ in *I2cm* or O_5_–O_4_–O_5_ in *Cmce*) is approximately the same, 160.60 and 160.44° for *I2cm* and *Cmce*, respectively. As such, the contraction of the NdTa_2_O_6_ layer does not arise from a simple change in the tilting of the TaO_6_ octahedra, as one might expect from an RUM perspective. Instead, the *I2cm* to *Cmce* structural transition involves significant distortions within the octahedra. The Ta–O_ax–L_ bond shows a noticeable decrease from 2.344 Å for *I2cm* (Ta–O_4_) to 2.306 Å for *Cmce* (Ta–O_4_), while the Ta–O_ax–S_ bond length and the O_ax–S_–Ta–O_ax–L_ bond angle remain essentially unchanged [1.795 (1) vs 1.798 (1) Å and 178.85 vs 177.04°, respectively]. The overall O_ax–S_ – O_ax–L_ distance therefore decreases from 4.138 (1) to 4.103 (1) Å (−0.9 %). While the TaO_6_ octahedra retain their SOJT distortion in the *Cmce* structure, its magnitude is decreased (0.16 to 0.14 Å) and the reduction in Ta–O_ax–L_ and Ta–O_eq_ (average reduces from 2.02 to 2.01 Å) bond lengths, leads to a reduction in octahedral volume. This is reflected in the Ta BVS increasing from 4.80 to 4.87. NTE due to polyhedral regularisation has been observed in other systems.^64^

Of course, the change in the structure of the TaO_6_ octahedra will be closely linked to changes in the Nd and Rb coordination environments, and the need to satisfy A site and oxygen bonding requirements will influence the specific distortion and tilts of TaO_6_ octahedra. In the polar ground state (*I2cm*), the Nd site has a favorable coordination environment, as indicated by a BVS of 2.75, which is achieved by the combination of an out-of-plane rotation along the *b*-axis, as well as the in-plane rotation along the *c*–axis coupled with a secondary polar displacement of the Nd ions in the *ab* plane. In the *Cmce* structure, which has antipolar displacements of the Nd ions, the Nd site coordination is very slightly lower (BVS reduced from 2.75 to 2.71). While there is a small increase in the average of Rb–O bond length (from 3.17 to 3.19 Å), resulting in a BVS change of 0.78 to 0.73, the expansion of the *ab* plane (∼+0.8 %) means that the Rb–O layer expansion along the *c-*axis is only ∼+0.53 % and doesn′t compensate for the larger contraction (∼−0.91 %) of the NdTa_2_O_6_ layers. These same effects are reflected in the average metal–metal distances reported in Table 4. When RNTO transforms from the *I2cm* to the *Cmce* phase, a significant reduction in average distance is observed for Ta–Nd (from 3.587 to 3.572 Å) and axial Ta–Ta (from 4.615 to 4.572 Å), while the Ta–Rb distance increases (from 4.320 to 4.347 Å). Thus interestingly we found that the uniaxial NTE in RNTO arises predominantly from the contraction of the NdTa_2_O_6_ layer, which is closely linked to the reduction in polyhedral distortions (Ta and Nd) coupled with reduced Ta-off-centering due to Nd displacement, while the change in the magnitude of octahedral tilting has minimal impact.

Our Raman analysis is consistent with these structural changes and the associated NTE, and it reflects the increased bond stiffness during the structural transition from *I2cm* to *Cmce*. Specifically, the mode hardening observed in the octahedral assymmetric vibration (586 cm^–1^) suggests an increase of the Ta–O bond strength in the *ab* plane across the transition. Additionally,the hardening of the translational mode of Nd (211 cm^–1^) and the mode of octahedral librations due to the Nd displacement (349 cm^–1^), indicates that the corresponding vibrations are more constrained following the phase transition from *I2cm* to *Cmce*. This would be triggered from the two octahedral rotations occurring during the phase transition [around *c*-axis *c* ^+^ to *c* ^–^ and around *b*–axis *a* ^–^ *b* ^–^ to *a* ^–^ *b* ^0^] and the associated cation displacements, leading to polyhedral distortion consistent with the *Cmce* phase. Hence, the transformation from the more distorted and flexible NdTa_2_O_6_ layer in the *I*2*c*m phase to the less distorted and more rigid/constraint NdTa_2_O_6_ layer in the *Cmce* phase is responsible for the observed mode hardening during the phase transition and the associated uniaxial NTE.

NTE in layered perovskites have been extensively studied in RP type oxides,^46,47,66,70–72^ however, our investigation provides insights into the origin of uniaxial NTE in DJ type oxides. We demonstrate that the observed uniaxial NTE is not driven by changes in TaO_6_ rotations, but is associated with the high flexibility of the NdTa_2_O_6_ intralayer bonds, and the degreee of freedom associated with the presence of the SOTJ ion (Ta^5+^, d^0^ cation) that is coupled to the neighboring Nd displacements (Figure 15). The transition from polar to antipolar displacement of the Nd ion, results in a decrease in the off-centering of the Ta within the TaO_6_ polyhedra and contraction of the NdTa_2_O_6_ layer, unlike RP systems, where the contraction of the unit cell occurs in the rock-salt layer via a completely distinct mechanism.^46,66,70^

**Figure 15:**
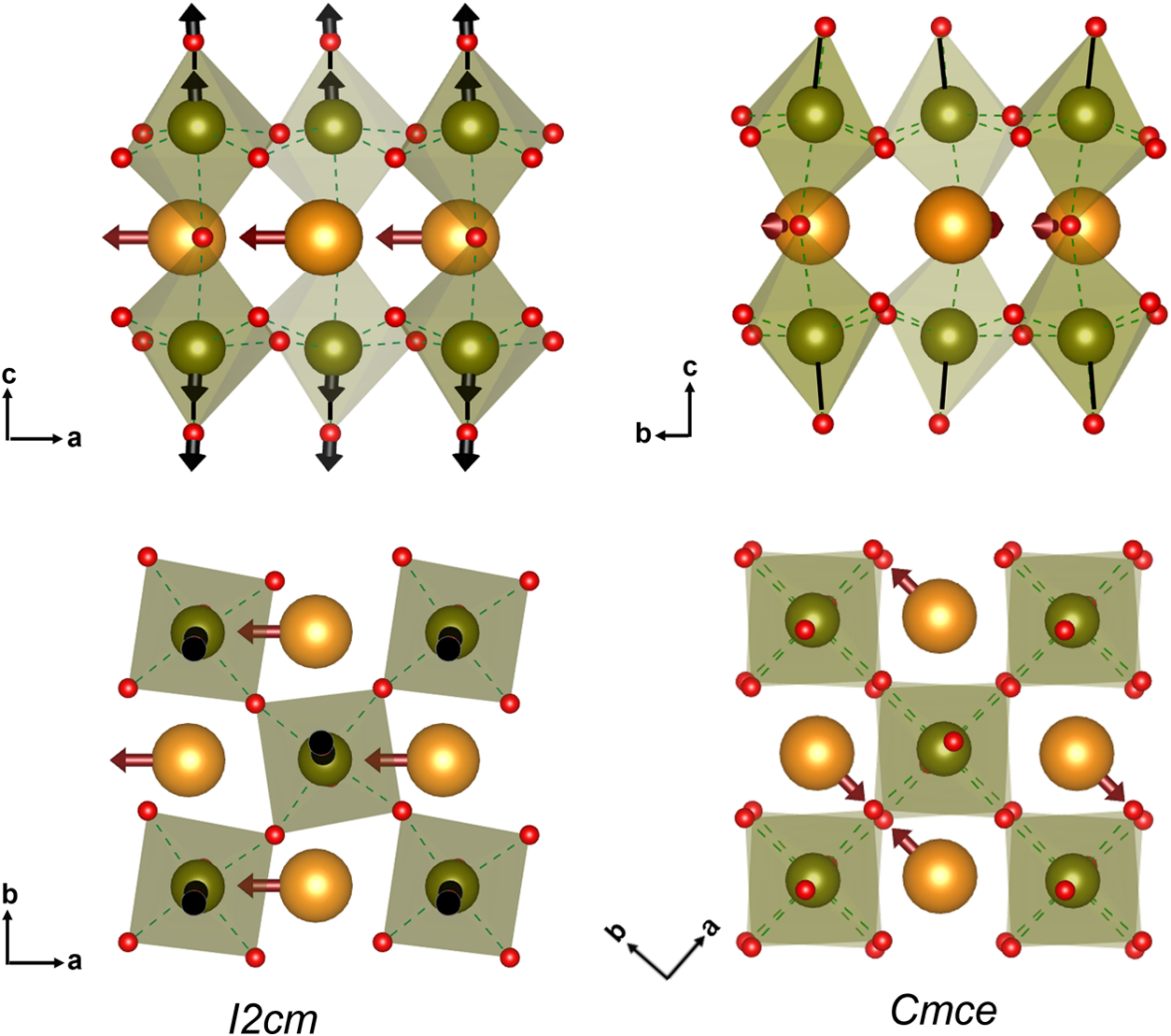
Representation of high flexibility of the NdTa_2_O_6_ intralayer bonds, the Nd-displacement directions and the Ta-off centerings with neighboring apical oxygen (rigidly bonded), in *I*2*c*m (left panel) and *Cmce* (right panel). Dotted green lines represent the flexible Ta–O bonds, and solid black lines represent the rigid Ta–O bonds. Black arrows represent the paired displacement of Ta and apical oxygens leading to expansion of NdTa_2_O_6_ layer in the polar *I*2*c*m symmetry at lower temperature and the brown arrows the orientation of Nd ions displacements.

The preference for polar ground structures in DJ oxides, specifically *I*2*cm* and *P*2_1_ *am*, depends on the A (rare earth) and A′ cations,^22^ with the relative stability of polar phases showing that Nb-based oxides are more stable than Ta-based ones, despite Nb^5+^ and Ta^5+^ having similar ionic radii and both exhibiting SOJT behavior.^21^ Our investigation aligns with this observation, suggesting that the presence of a stronger SOJT ion, such as Nb^5+^, which exhibits a stronger SOJT effect than Ta^5+^ due to its greater covalent bonding tendency with oxygen,^73^ also influences overall structural distortions, including octahedral rotations/deformations, and promotes A-site displacements, thereby contributing to the stability of polar ground-state structures.

Additionally, during the first-order transition, we observe an expansion of the Rb–O layer (∼+0.53 %) alongside the reduction in the thickness of the NdTa_2_O_6_ layer (∼−0.90 %). This suggests a potential competition between the extent of thermal expansion and compression behaviors of the A′–O layer and AB_2_O_6_ layers, influencing the mechanism of uniaxial NTE in DJ type oxides. The specific thermal response strongly depends on the nature of the A′ and B-site ions, which determine how each layer expands or contracts with temperature. Thus it would be interesting to study the thermal expansion behavior of other DJ type oxides to further explore the critical role of different metal ions in driving thermal expansion/compression across the first-order phase transition.

### Electronic Properties

The electronic partial density of states (PDOS) plots are shown in Figure 16 for all four studied structures. The valence band maximum (VBM) is mostly composed of O-p states (which is common for the majority of transition-metal and rare-earth oxides). At lower energies of the valence band, some hybridization is shown between the Ta-d and O-p states; and at around −2 eV a resonant peak appears and defines the occupied magnetic f states of Nd. The minimum of the conduction band (CBM) is composed of very shallow Ta-d states, and for slightly increasing energies a mixture with the Nd-f (with a resonant peak) with the Ta-d states is observed. The O states are those that evidence more differences between the four structural systems, and would therefore correspond to the distortions of the perovskite cages accordingly, as symmetry decreases, and very similar to what occurs to RP oxides.^74^

**Figure 16:**
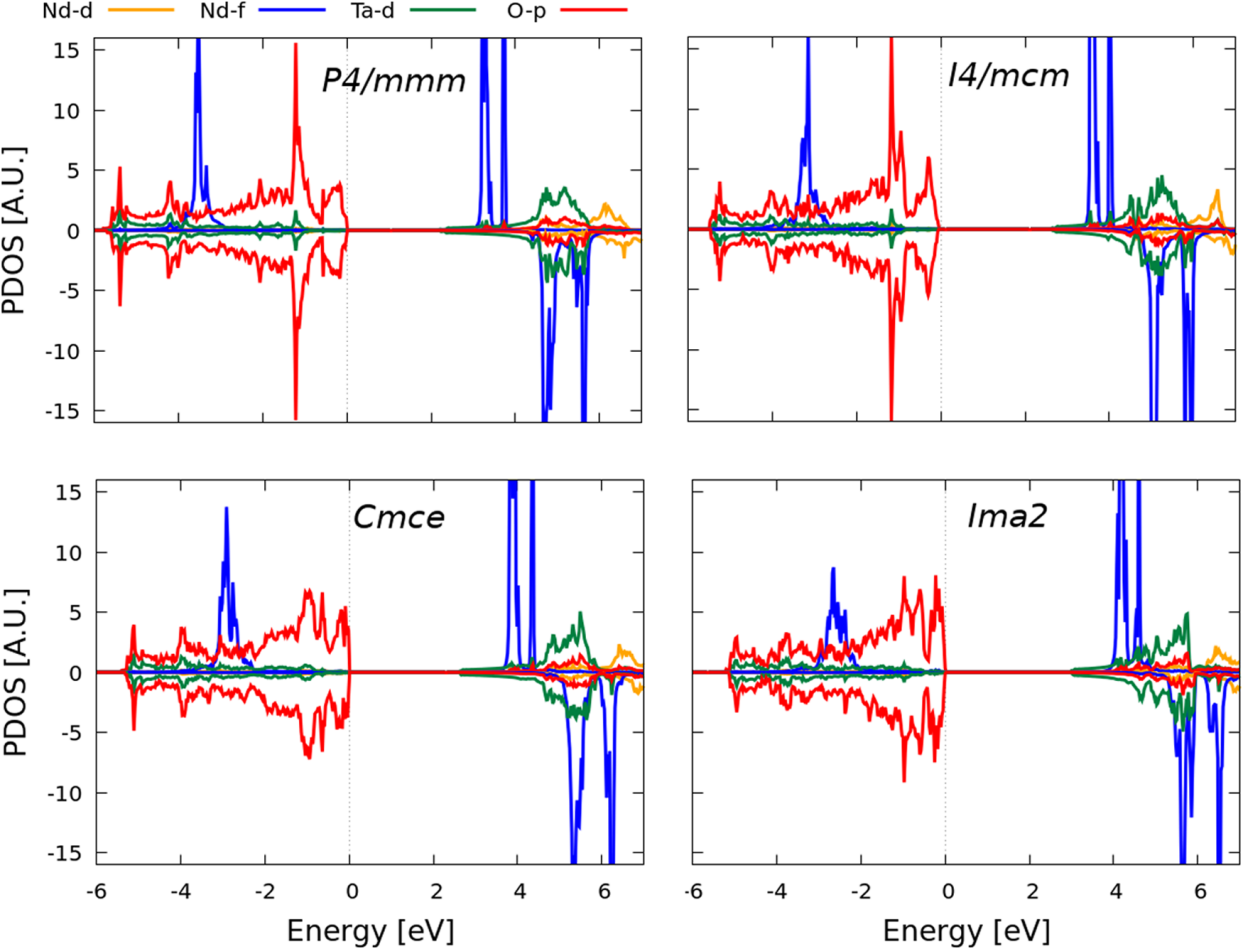
Partial density of states (per formula unit) of the four studied polymorphs by considering *U*_eff_ = 6 eV, and the high-spin magnetic ordering. The VBM was aligned at 0 eV.

We present in Table 5, the values of the electronic band gap and effective masses of the charge carriers of the ground-state structure, when considering the high-spin ordering. The electronic band gap of the *I2cm* system evidence an indirect character, with a width of around 2.97 eV (Figure 17). We have to state that the direct gap is 3.00 eV (along the $\overset{®}{{\mathrm{XU}}_{2}\;}$ segment). From DFT + *U* calculations and due to the observed mild differences in energies of the CBM, such features will not allow us to provide a definitive conclusion about the nature/character of the energy band gap.

**Figure 17:**
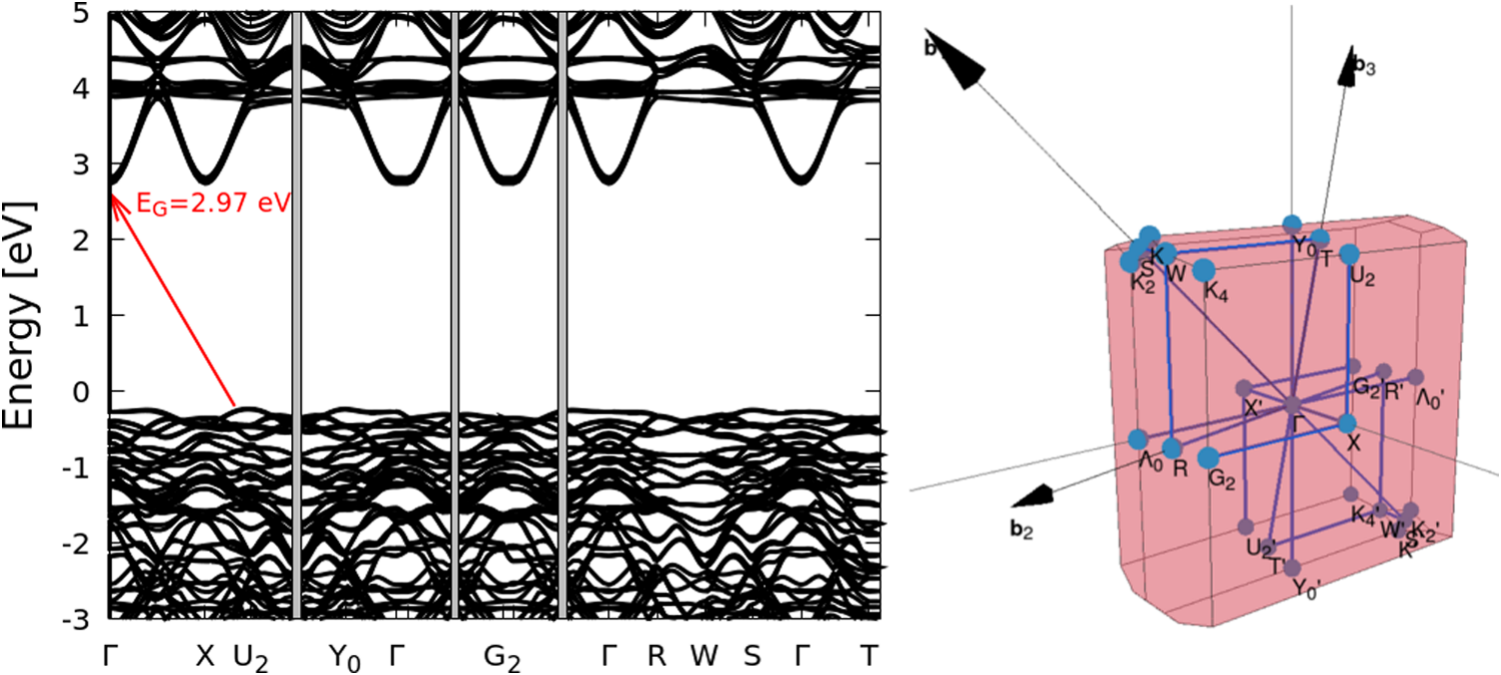
Electronic band-structure of the *I2cm* polymorph (left panel). BZ and special BZ points representation for *I2cm* (right panel) and resorting to the SEEKPATH online tool to obtain the high-symmetry pathway.^75,76^

**Table 5:** Band Gap and Effective Masses of the *I2cm* Polymorph (S.G. 46) of RNTO, Considering *U*_eff_ = 6 eV^a^

	Effective Masses	
Band gap (eV)	m*_h_	m*_e_
2.97 (indirect)	–2.903 $\left(\overset{®}{{\mathrm{XU}}_{2}\;}\to X\right)$	0.523 (Γ → *X*)
3.00 (direct)		0.578 (Γ → *Y*_0_)
		0.509 (Γ → *R*)
		0.610 (Γ → *S*)
		0.531 (Γ → *T*)

a Values are given in units of the mass of the electron (*m*_0_ = 9.11 × 10^–31^ kg).

Comparing with results from ref 29., our DFT + *U* band gap width is below to what had been experimentally estimated with a value of 4.2 eV.^29^ Since DFT is known to underestimate the absolute value of the band gap, and the underestimation is expected to be similar in all structures, such a feature will not affect the systematic discussion of results. Calculations by employing the hybrid functional (i.e., HSE06) could provide a closer estimate to the experimental value; however, and due to the characteristics of the studied material, such calculations would prove to be quite costly, CPU wise, and therefore this analysis was not considered for the present work.

Among the studied RbLnTa_2_O_7_ (Ln = La, Pr, Nd and Sm) catalysts, it has been evidenced that RbNdTa_2_O_7_ shows the highest photocatalytic activity to decompose water into *H*_2_ and *O*_2_, under UV-light irradiation.^77,78^ This may imply that excitation of the 4f states contributes to the photocatalytic features of the layered perovskite tantalates.^29,79^

Moreover, another property of interest to further explore is the effective masses, since respective features are also relevant to infer about the mobility of the charge carriers. Since we observe a more parabolic dispersion of the conduction bands of the ground-state structures (Figure 17), a smaller electron effective mass, m*_e_, will be observed, and thus electron migration is favored. On the other hand, since the valence bands are more flat, the hole effective mass, m*_h_ will be heavier. We can observe in Table 5 for *I2cm*, that indeed while the electron effective mass is m*_e_ ∼ 0.5–0.6 m_0_ around the high-symmetry Γ point (CBM), the hole effective mass (VBM) is considerably more heavy with m*_h_ = −2.9 *m*_0_.

## Conclusions

This work provides a deeper insight into the uniaxial NTE in RNTO through a combination of experimental techniques such as PXRD, NPD and Raman spectroscopy, along with DFT + *U* calculations. The uniaxial NTE observed perpendicular to the layers of RNTO, when *I2cm* (hybrid improper ferroelectric) transitions to *Cmce* (antipolar), is dominated by compression of the NdTa_2_O_6_ layers, coupled with changes in Ta-off-centering and Nd-ion displacements, with minimal impact from octahedral bilayer tilts and limited to the temperature range where the first-order structural transition occurs. This phase transition driven uniaxial NTE, is associated with the high flexibility of the NdTa_2_O_6_ intralayer bonds, highlighting the intricate interplay of the presence of SOJT Ta^5+^ ion, Nd-ion displacement, Nd*/*Ta-ion coordination and polyhedral regularisation. Our temperature-dependent Raman studies further confirm the first-order structural phase transition and also support that the observed NTE in RNTO is related to the contraction of the NdTa_2_O_6_ layer (hardening of modes across the phase transition for the in-plane octahedral vibrations and those coupled with Nd displacement). In addition to this, we have identified the previously unknown high-symmetry phase transition temperature as *T*_3_ ∼ 1150 K, where the structure transforms from *I*4/*mcm* → *P*4/*mmm*.

From our DFT + *U* calculations, we have obtained an energetic equivalent structure to *I2cm*, which is a *Pc2*_1_ *n* polymorph, meaning that both systems may coexist at the low-temperature regime. The electronic PDOS were probed for the four experimentally observed polymorphs, while for the band dispersions we solely calculated for the lowest energetic structure (*I2cm*), mainly to analyze the band gap width and respective (in)­direct character, as well as the effective masses of the charge carriers. The PDOS show that the VBM is mostly composed by the O p-states, whereas the CBM by the Ta d-states, and in accordance to other oxide families. Differences are evidenced among the phases, mostly of the O states, where a resonant peak at the valence band (slightly above −2 eV) tends to disappear as the symmetry is broken toward lower phases. This is consistent with the octahedral TaO_6_ distorting as the symmetry breaks from *P*4/*mmm* toward *I2cm*. With respect to the band structure, we observe that the indirect and the direct gap is around ∼3 eV, and it is challenging for us to determine the character of the gap under the DFT + *U* framework. The electron effective masses are found to be very dispersive, being much lighter than the hole effective masses. Such effects may provide useful applications, as is the case for n-type transparent conducting oxides under useful doping conditions.

Our insights highlight the uniaxial NTE in RNTO, emphasizing the critical role of metal ion coordination, influence of SOJT ion (Ta^5+^) and polar to antipolar displacement of Nd ions in driving perovskite layer compression during the hybrid improper ferroelectric to antipolar phase transition. These findings open opportunities to explore in detail the thermal expansion and lattice dynamical study on DJ oxides and to provide chemical control on the mechanism driving NTE. Future research could focus on designing novel materials or modifying existing ones to enhance the NTE, offering significant promise for both fundamental studies and practical applications, particularly in enabling the direct conversion of thermal energy into mechanical motion. Additionally, RNTO opens up possibilities in advanced materials research as a photocatalytic and photovoltaic material through appropriate band gap engineering.

## Supplementary Material



## Notes

Rietveld refinement structural parameters for RbNdTa_2_O_7_ from neutron powder diffraction data collected at different temperatures; the lattice parameters obtained from DFT + *U* for all the structures; deconvoluted Raman spectra for RbNdTa_2_O_7_ obtained at 300 K (PDF)
