# Supplementary material for: Structural Distortions and Uniaxial Negative Thermal Expansion in the Polar Dion–Jacobson Oxide RbNdTa2O7
Source: Chem Mater. 2025 May 15;37(15):5529–46. doi: 10.1021/acs.chemmater.5c00137 (PMC12821136; doi:10.1021/acs.chemmater.5c00137)
Supplement: Supplementary file 1 [file cm5c00137_si_001.pdf]

Structural Distortions and Uniaxial Negative Thermal Expansion in the Polar Dion-Jacobson Oxide $RbNdTa_2O_7$

P. Neenu Lekshmi,[*,†] E. Lora da Silva,[†,‡] P. Rocha-Rodrigues,[†] John S. O. Evans,[¶] João Horta Belo,[†] Pedro Silva de Sousa,[†] Alicia María Manjón-Sanz,[§] António M. dos Santos,[§] Armandina M. L. Lopes,[†] and João Pedro Araújo[*,†]

†*IFIMUP, Institute of Physics for Advanced Materials, Nanotechnology and Photonics, Department of Physics and Astronomy, Faculty of Sciences, University of Porto, Rua do Campo Alegre, 687, 4169-007 Porto, Portugal*

‡*High Performance Computing Chair, University of Évora, Rua Romão Ramalho 59, 7000-671 Évora, Portugal*

¶*Department of Chemistry, University Science Site, Durham University, South Road, Durham, DH1 3LE, UK*

§*Neutron Scattering Division, Oak Ridge National Laboratory, Oak Ridge, Tennessee 37831, USA*

E-mail: neenulekshmi@fc.up.pt; jearaujo@fc.up.pt

Supporting Information

Rietveld refinement structural parameters for $RbNdTa_2O_7$ from neutron powder diffraction data collected at different temperatures

Table S1: At 300 K for *I2cm* and *Pc2$_1$n* models

*I2cm S.G.*46 - Lattice parameter (Å): a = 5.4313 (1); b =5.4239 (1); c =22.1623 (4) Volume (Å^3) = 652.88 (2) ;R$_{wp}$= 2.51;χ2 =4.68						
Atom	Site	x	y	z	Occ.	U$_i$*so* (Å^2)
Rb	*4a*	0.7447 (1)	0	0	1	0.017
Nd	*4b*	0.7291 (1)	0.0052 (1)	0.2500 (1)	1	0.012
Ta	*8c*	0.2526 (1)	0.9995 (1)	0.3526 (1)	1	0.010
O1	*8c*	0.9823 (4)	0.7767 (1)	0.3238 (1)	1	0.021
O2	*8c*	0.0571 (1)	0.2990 (1)	0.3451 (1)	1	0.018
O3	*8c*	0.7435 (1)	0.5487 (1)	0.4322 (1)	1	0.020
O4	*4b*	0.7715 (1)	0.4335 (1)	0.2500 (1)	1	0.017
*Pc2$_1$n S.G.*33- Lattice parameter (Å): a = 5.4241 (1); b =5.4314 (1); c =22.1617 (5) Volume (Å^3) = 652.89 (3) ;R$_{wp}$= 2.63 ;χ2 =5.24						
Atom	Site	x	y	z	Occ.	U$_i$*so* (Å^2)
Rb	*4a*	0.7562 (1)	0.2747 (1)	0.7520 (1)	1	0.0343 (1)
Nd	*4a*	0.7574 (1)	0.2918 (1)	0.0037 (1)	1	0.0101 (1)
Ta1-1	*4a*	0.7468 (1)	0.7629 (1)	0.1033 (1)	1	0.0069 (1)
Ta1-2	*4a*	0.2479 (1)	0.2691 (1)	0.6020 (1)	1	0.0136 (1)
O1-1	*4a*	0.5243 (1)	0.0483 (1)	0.0768 (1)	1	0.0322 (1)
O1-2	*4a*	0.0397 (4)	0.52093 (1)	0.5710 (1)	1	0.0153 (1)
O2-1	*4a*	0.0471 (1)	0.9597 (1)	0.0948 (1)	1	0.0286 (1)
O2-2	*4a*	0.5475 (1)	0.4650 (1)	0.5950 (1)	1	0.0138 (1)
O3-1	*4a*	0.2901 (1)	0.2521 (1)	0.1838 (1)	1	0.036 (1)
O3-2	*4a*	0.8024 (1)	0.7835 (1)	0.6810 (1)	1	0.0112 (1)
O4	*4a*	0.1836 (1)	0.2451 (1)	0.0012 (1)	1	0.0159 (1)

Occ. - site occupancy; U$_{iso}$ - isotropic thermal parameter.

Table S2: At different temperatures with the best structural models

600 K - *Cmce S.G.*64 - Lattice parameter (Å): a = 7.7029 (3); b =7.7217 (3); c =22.2209 (7) Volume (Å^3)=1321.69 (8); R$_{wp}$=2,18 ; χ2=4.60						
Atom	Site	x	y	z	Occ.	U$_{i}$*so* (Å^2)
Rb	*8f*	0	0.2531 (1)	0.2589 (1)	1	0.041 (1)
Nd	*8f*	0	0.2479 (1)	0	1	0.021 (1)
Ta	*16g*	0.7496 (1)	0.0014 (1)	0.1013 (1)	1	0.009 (1)
O1	*8f*	0	0.4680 (1)	0.5940 (1)	1	0.024 (1)
O2	*8f*	0	0.4989 (1)	0.0727 (1)	1	0.037 (1)
O3	*16g*	0.2323 (1)	0.2479 (1)	0.9190 (1)	1	0.045 (1)
O4	*8d*	0.7857 (1)	0	0	1	0.028 (1)
O5	*16g*	0.7224 (1)	0.9917 (1)	0.1821 (1)	1	0.031 (1)
800 K - *Cmce S.G.*64 - Lattice parameter (Å): a = 7.7214 (4); b =7.7330 (3); c =22.2893 (7) Volume (Å^3)=1330.88 (9); R$_{wp}$=2.26; χ2 =4.07						
Atom	Site	x	y	z	Occ.	U$_{i}$*so* (Å^2)
Rb	*8f*	0	0.2531 (1)	0.2589 (1)	1	0.049 (1)
Nd	*8f*	0	0.2479 (1)	0	1	0.023 (1)
Ta	*16g*	0.7496 (1)	0.0014 (1)	0.1013 (1)	1	0.009 (1)
O1	*8f*	0	0.4680 (1)	0.5940 (1)	1	0.018 (1)
O2	*8f*	0	0.4989 (1)	0.0727 (1)	1	0.047 (1)
O3	*16g*	0.2323 (1)	0.2479 (1)	0.9190 (1)	1	0.044 (1)
O4	*8d*	0.7857 (1)	0	0	1	0.030 (1)
O5	*16g*	0.7224 (1)	0.9917 (1)	0.1821 (1)	1	0.039 (1)
970 K - *I4/mcm S.G.*140 - Lattice parameter (Å): a = b =5.4737 (1); c =22.3480 (9) Volume (Å^3)=669.59(3); R$_{wp}$=2.23; χ2=5.04						
Atom	Site	x	y	z	Occ.	U$_{i}$*so* (Å^2)
Rb	*4d*	0	0.5000	0	1	0.063 (1)
Nd	*4b*	0	0.5000	0.2500	1	0.029 (1)
Ta	*16l*	0	0	0.1013 (1)	1	0.008 (1)
O1	*8f*	0.7256 (1)	0.2256 (1)	0.3340 (1)	1	0.042 (1)
O2	*4a*	0	0	0.9319 (1)	1	0.056 (1)
O3	*16g*	0	0	0.2500	1	0.062 (1)
1000 K - *I4/mcm S.G.*140 - Lattice parameter (Å): a = b =5.4758 (1); c =22.3571 (1) Volume (Å^3)=670.37 (1); R$_{wp}$=2.52; χ2=7.82						
Atom	Site	x	y	z	Occ.	U$_{i}$*so* (Å^2)
Rb	*4d*	0	0.5000	0	1	0.065 (1)
Nd	*4b*	0	0.5000	0.2500	1	0.027 (1)
Ta	*16l*	0	0	0.8517 (1)	1	0.008 (1)
O1	*8f*	0.7264 (1)	0.2264 (1)	0.3339 (1)	1	0.042 (1)
O2	*4a*	0	0	0.9319 (1)	1	0.058 (1)
O3	*16g*	0	0	0.2500	1	0.060 (1)

Table S3: Continue

Atom	Site	x	y	z	Occ.	U_{iso} (Å^2)
1150 K - *P4/mmm S.G.*123 - Lattice parameter (Å): a = b =3.8803 (1); c =11.2000 (3) Volume (Å^3)=168.673 (8); R_{wp}=1.86; χ2=8.04						
Atom	Site	x	y	z	Occ.	U_{iso} (Å^2)
Rb	*1b*	0	0	0.5000	1	0.074 (1)
Nd	*1a*	0	0	0	1	0.029 (1)
Ta	*2h*	0.5000	0.5000	0.8517 (1)	1	0.012 (1)
O1	*4i*	0	0.5000	0.1649 (1)	1	0.050 (1)
O2	*2h*	0.5000	0.5000	0.3625 (1)	1	0.063 (1)
O3	*1c*	0.5000	0.5000	0	1	0.065 (1)
1200 K - *P4/mmm S.G.*123 - Lattice parameter (Å): a = b =3.8823 (1); c =11.2077 (1) Volume (Å^3)=168.930 (7); R_{wp}=2.47; χ2=7.93						
Atom	Site	x	y	z	Occ.	U_{iso} (Å^2)
Rb	*1b*	0	0	0.5000	1	0.084 (1)
Nd	*1a*	0	0	0	1	0.029
Ta	*2h*	0.5000	0.5000	0.2033 (1)	1	0.012
O1	*4i*	0	0.5000	0.1668 (1)	1	0.056 (1)
O2	*2h*	0.5000	0.5000	0.3637 (1)	1	0.070 (1)
O3	*1c*	0.5000	0.5000	0	1	0.076 (1)
1275 K - *P4/mmm S.G.*123 - Lattice parameter (Å): a = b =3.8850 (1); c =11.2217 (4) Volume (Å^3)=169.374 (8); R_{wp}=2.68; χ2=4.52						
Atom	Site	x	y	z	Occ.	U_{iso} (Å^2)
Rb	*1b*	0	0	0.5000	1	0.083 (1)
Nd	*1a*	0	0	0	1	0.030 (1)
Ta	*2h*	0.5000	0.5000	0.2029 (1)	1	0.011 (1)
O1	*4i*	0	0.5000	0.1672 (1)	1	0.053 (1)
O2	*2h*	0.5000	0.5000	0.3625	1	0.069 (1)
O3	*1c*	0.5000	0.5000	0	1	0.067 (1)

Occ. - site occupancy; U_{iso} - isotropic thermal parameter.

Table S4: Rietveld Refinement Structural Parameters at 1100 K - Two phase model: *I4/mcm* and *P4/mmm*

1100 K - *I4/mcm* *S.G.*140 - Lattice parameter (Å): a = b =5.4847 (1); c =22.4065 (1) Volume ($Å^3$)=674.04 (1); Phase Fraction= 47.15 %						
Atom	Site	x	y	z	Occ.	U_iso ($Å^2$)
Rb	*4d*	0	0.5000	0	1	0.071 (1)
Nd	*4b*	0	0.5000	0.2500	1	0.029 (1)
Ta	*16l*	0	0	0.8515 (1)	1	0.007 (1)
O1	*8f*	0.7306 (1)	0.2306 (1)	0.3339 (1)	1	0.040 (1)
O2	*4a*	0	0	0.9317 (1)	1	0.061 (1)
O3	*16g*	0	0	0.2500	1	0.063 (1)
1100 K - *P4/mmm* *S.G.*123 - Lattice parameter (Å): a = b =3.8768 (1); c =11.1805 (1) Volume ($Å^3$)=168.04 (1); Phase Fraction=51.01 %						
Atom	Site	x	y	z	Occ.	U_iso ($Å^2$)
Rb	*1b*	0	0	0.5000	1	0.076 (1)
Nd	*1a*	0	0	0	1	0.030 (1)
Ta	*2h*	0.5000	0.5000	0.2031 (1)	1	0.010 (1)
O1	*4i*	0	0.5000	0.1669 (1)	1	0.051 (1)
O2	*2h*	0.5000	0.5000	0.3635	1	0.061 (1)
O3	*1c*	0.5000	0.5000	0	1	0.064 (1)

R_{wp}=1.80; $\chi 2$=6.94; Occ. - site occupancy; U_{iso} - isotropic thermal parameter.

Lattices Parameters

The lattice parameters obtained from DFT+U, and for the five studied structures are shown in Table. S5. These are compared to experimental data obtained through NPD, and to the temperatures to which each structure was measured.

Table S5: Lattice parameters of the five polymorphs of RNTO and respective volumes, calculated from DFT+U (U_{eff}=6 eV) and comparison is made from experimental neutron powder diffraction data (shaded cells).

Phase	Lattice parameters (Å)			Volume (Å^3)
	a_0	b_0	c_0	
P4/mmm	3.90	3.90	11.33	172.47
	3.88	3.88	11.20	168.93
I4/mcm	5.45	5.45	22.94	681.79
	5.47	5.47	22.35	670.39
Cmce	7.80	7.82	22.48	1371.46
	7.70	7.72	22.22	1321.64
I2cm	5.47	5.49	22.59	678.58
	5.43	5.42	22.16	652.88
$Pc2_1n$	22.61	5.46	5.49	678.70
	22.16	5.43	5.42	652.89

We can observe that the DFT+U parameters are within the error margin that is normally observed when comparison of the structural parameters are considered with experimental data. However, the ground-state structures are those that show larger differences in the volume parameters, when comparison is considered with the NPD data at room temperature. For these two ground-state structures, *I2cm* and $Pc2_1n$, the a_0 and b_0 lattice parameters are quite close to those of NPD (with a mild increase of values), while the c_0 parameter is slightly increased with respect to experimental data, thus overestimating the volume of both unit cells by ∼4%.

As for the *Cmce* structural phase, the major difference is also observed for the the c_0 lattice parameter, although this is more mild, since the relative difference between the computational and experimental volumes are only of 1.7%.

As temperature increases with the structure transitioning to the *I4/mcm* crystal, we

observe an inverse trend of the a_0 and b_0 parameters, since these are relatively smaller than the experimental values; however, c_0 is increased. This reversal of results, provides a theoretical value of the volume quite close to the NPD data, where the variation is only of $\sim$1.7%.

Finally the *P4/mmm* again shows the theoretical lattice parameters being slightly higher than experimental results, thus increasing the volume with $\sim$2%.

Deconvoluted Raman Spectra for $RbNdTa_2O_7$ obtained at 300 K

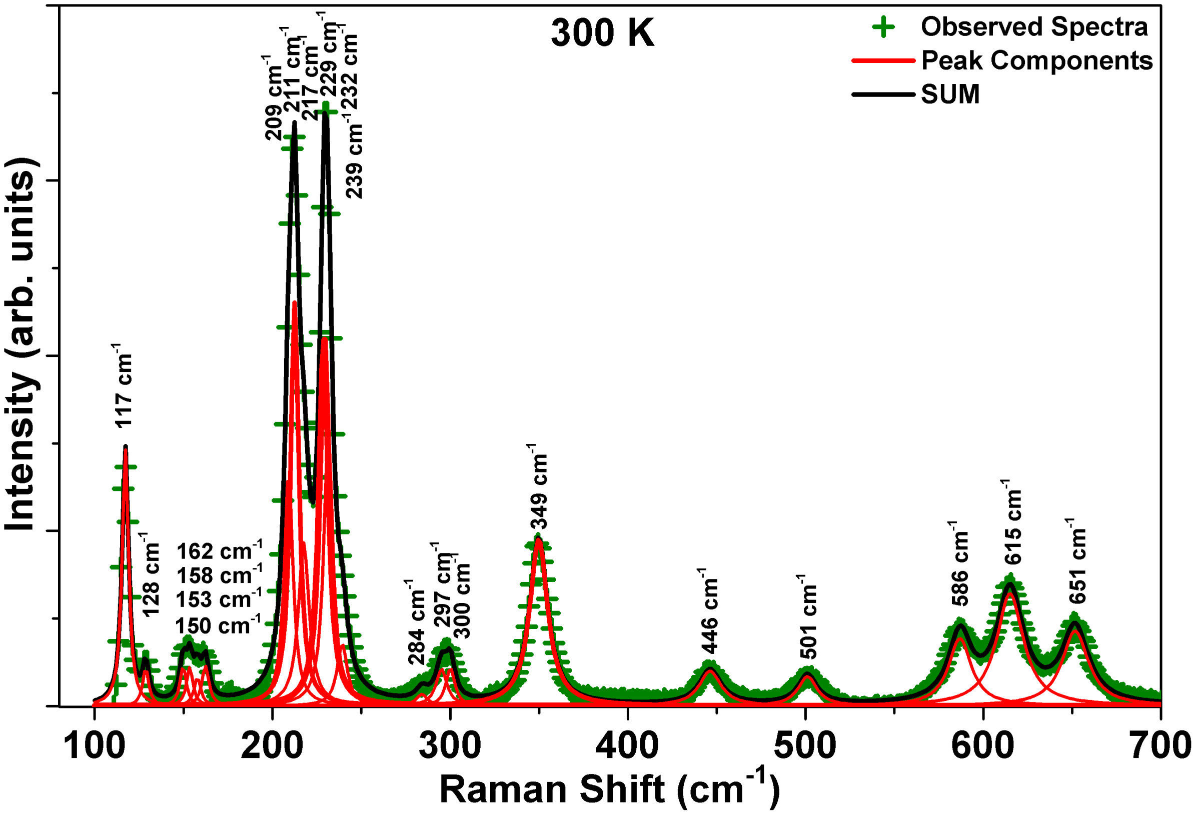

Figure S1: Deconvoluted Raman Spectra obtained at 300 K
